# CCR7 as a novel therapeutic target in t-cell PROLYMPHOCYTIC leukemia

**DOI:** 10.1186/s40364-020-00234-z

**Published:** 2020-10-24

**Authors:** Carlos Cuesta-Mateos, Patricia Fuentes, Alexandra Schrader, Raquel Juárez-Sánchez, Javier Loscertales, Tamara Mateu-Albero, Lorena Vega-Piris, Marina Espartero-Santos, Ana Marcos-Jimenez, Blanca Andrea Sánchez-López, Yaiza Pérez-García, Dennis Jungherz, Sebastian Oberbeck, Linus Wahnschaffe, Anna Kreutzman, Emma I. Andersson, Satu Mustjoki, Edgar Faber, Ana Urzainqui, Manuel Fresno, Kostantino Stamatakis, Arantzazu Alfranca, Fernando Terrón, Marco Herling, María Luisa Toribio, Cecilia Muñoz-Calleja

**Affiliations:** 1 Immunology Department, Hospital Universitario de La Princesa, IIS-IP, C/ Diego de León 62, 28006 Madrid, Spain; 2 IMMED S.L., Immunological and Medicinal Products, Madrid, Spain; 3 Immune System Development and Function Unit, Centro de Biología Molecular Severo Ochoa, CSIC-UAM, Madrid, Spain; 4 Department I of Internal Medicine, Center for Integrated Oncology (CIO) Aachen-Bonn-Cologne-Duesseldorf (ABCD), Cologne Cluster of Excellence in Cellular Stress Response and Aging-Associated Diseases (CECAD), and Center of Molecular Medicine Cologne (CMMC), The University of Cologne, Cologne, Germany; 5 Hematology Department, Hospital Universitario de La Princesa, IIS-IP, Madrid, Spain; 6 Methodology Unit, Hospital Universitario de La Princesa, IIS-IP, Madrid, Spain; 7 Department of Hematology, Hematology Research Unit Helsinki, Helsinki University Hospital Comprehensive Cancer Center, Helsinki, Finland; 8 Translational Immunology Research Program and Department of Clinical Chemistry, University of Helsinki, Helsinki, Finland; 9 Department of Hemato-Oncology, Faculty Hospital Olomouc, Faculty of Medicine and Dentistry Palacky University, Olomouc, Czech Republic; 10 Department of Cell Biology and Immunology, Centro de Biología Molecular Severo Ochoa, CSIC-UAM, Madrid, Spain; 11 Universidad Autónoma de Madrid, Madrid, Spain

**Keywords:** CCR7, T-PLL, mAb, T-cell lymphomas, Immunotherapy

## Abstract

T-cell prolymphocytic leukemia (T-PLL) is a poor prognostic disease with very limited options of efficient therapies. Most patients are refractory to chemotherapies and despite high response rates after alemtuzumab, virtually all patients relapse. Therefore, there is an unmet medical need for novel therapies in T-PLL. As the chemokine receptor CCR7 is a molecule expressed in a wide range of malignancies and relevant in many tumor processes, the present study addressed the biologic role of this receptor in T-PLL. Furthermore, we elucidated the mechanisms of action mediated by an anti-CCR7 monoclonal antibody (mAb) and evaluated whether its anti-tumor activity would warrant development towards clinical applications in T-PLL. Our results demonstrate that CCR7 is a prognostic biomarker for overall survival in T-PLL patients and a functional receptor involved in the migration, invasion, and survival of leukemic cells. Targeting CCR7 with a mAb inhibited ligand-mediated signaling pathways and induced tumor cell killing in primary samples. In addition, directing antibodies against CCR7 was highly effective in T-cell leukemia xenograft models. Together, these findings make CCR7 an attractive molecule for novel mAb-based therapeutic applications in T-PLL, a disease where recent drug screen efforts and studies addressing new compounds have focused on chemotherapy or small molecules.

**Supplementary information:**

**Supplementary information** accompanies this paper at 10.1186/s40364-020-00234-z.

## Background

T-cell prolymphocytic leukemia (T-PLL) is a rare hematological malignancy, which represents ~ 2% of all mature lymphocytic leukemia in adults [1, 2]. T-PLL is characterized by a rapid proliferation of mature post-thymic prolymphocytes [1, 3, 4] and the presence of cytogenetic abnormalities affecting the chromosomes 14q32 and Xq28 that lead to over-expression and activation of the *TCL1* and *MTCP1* oncogenes, respectively [5–7]. In addition, T-PLL is featured by an aggressive clinical course and by poor responses to alkylating chemotherapies [1, 4, 8, 9]. Therapeutic options for T-PLL have broadened with the advent of purine analogs [10], and particularly by the anti-CD52 monoclonal antibody (mAb) alemtuzumab [11, 12]. With these options, response rates exceed 90% and the median overall survival (OS) was extended from ~ 7.5 to ~ 20 months following alemtuzumab monotherapy or in combination with purine analogs [8, 10, 13–15]. Nevertheless, the relapse rate after these agents is ~ 100% with a median duration of remissions of ~ 12 months. Only 10–15% of patients experience long-term (> 5 years) survival [8, 13, 16, 17] after consolidation with allogeneic hematopoietic stem cell transplantation (allo-HSCT) [16, 18]. Given these unsustained responses after induction and the limited eligibility for a consolidating allo-HSCT, there is an urgent clinical need for more efficient and profound tumor cell clearance in T-PLL.

To overcome the restricted availability of active therapies in T-PLL, we focused on the homeostatic chemokine receptor CCR7 as a targetable structure. CCR7 controls the entry of normal naïve (T_N_) and central memory T-cells (T_CM_) into the secondary lymphoid organs (SLO). CCR7 is expressed in mature T-cell malignancies, such as adult T-cell leukemia/lymphoma (ATLL) [19] and Sézary syndrome (SS) [20], and enables the entry of acute lymphoblastic leukemia (ALL) cells to the central nervous system (CNS) where CCR7 promotes survival and proliferation [21, 22]. In the present work, we studied the expression and functions of CCR7 in primary samples of T-PLL and evaluated in vitro and in vivo its potential as a therapeutic target for a mAb-based therapy.

## Methods

### Samples

T-PLL patients included in this study were diagnosed according to WHO and refined consensus criteria [2, 3]. Informed consent was obtained in each contributing center in accordance with the Declaration of Helsinki. Experimental procedures were approved by the Institutional Board of the Hospital de La Princesa. Cells isolation from freshly donated peripheral blood (PB) was done using Ficoll-paque plus density gradient centrifugation (Amersham Biosciences, Little Chalfont, UK). Cells were cultured in RPMI-1640 media supplemented with 10% heat-inactivated fetal bovine serum (FBS), 2 mM L-glutamine and 100 U/mL penicillin/100 μg/mL streptomycin at 37 °C in 5% CO_2_. Peripheral blood mononuclear cells (PBMCs) from healthy donors (HD) were obtained from PB or blood buffy coats. Human umbilical vein endothelial cells (HUVEC) were isolated from freshly donated umbilical cords in accordance to the Declaration of Helsinki.

### Cell lines

The human cell line SUP-T11 was purchased from the DSMZ German collection of microorganisms and cell cultures (Braunschweig, Germany). Identity was confirmed using multiplex PCR of minisatellite markers performed by DSMZ. Cells were cultured according to supplier’s protocols. Absence of *Mycoplasma* contamination was routinely tested for with MYCOPLASMA Gel Form kit (Biotools, Madrid, Spain).

### Reagents

The antibody alemtuzumab was provided by Genzyme (Cambridge, MA). Mouse anti-hCCR7 mAb (150503 clone, IgG2a) and the respective isotype control (IC) were obtained from R&D Systems (Minneapolis, MN). The anti-CCR7 mAb was selected owed to its reported ability to block CCR7-ligand interactions and killing target cells [22–25]. In a confirmatory assay, clone 150503 showed no agonistic effects in β-arrestin recruitment assays whereas CCL21 triggered a strong activation (Supplementary Figure 1-A). Similarly, we confirmed that the selected clone did not induce internalization processes upon binding to surface CCR7 (Supplementary Figure 1-B).

### Flow cytometry

Expression levels of CCR7, CD52, and CCR4 on T-PLL cells were determined using PE (phycoerythrin)- or PE-cy5.5-conjugated anti-CCR7 (clone 150503, R&D Systems, Minneapolis, MN), PE-conjugated anti-CD52 or PE-conjugated anti-CCR4 (clones 4C8 and 1G1, respectively, BD Biosciences, San Jose, CA), with auxiliary CD5-FITC (clone UCHT2), CD7-APC (clone M-7 T01), and CD3-APC-H7 (clone SK7) (all from BD Biosciences). In all cases, an appropriate fluorochrome-conjugated IC was included and 10^4^ neoplastic T-cells were acquired. Immunofluorescence staining was analyzed on a FACS Canto II flow cytometer (BD Biosciences) using Infinicyt v.1.3.0 (Cytognos, Salamanca, Spain) and Diva v.2.4 (BD Biosciences) software. Results are presented as the percentage of CCR7 positive cells and the median fluorescence intensity (MFI) of CCR7, CD52, and CCR4 expression relative to the IC (RMFI). For determination of CCR7 expression on T_N_ or T_CM_, we used a flow cytometry panel containing the following antibodies: CCR7 PE (R&D), and CD45RA-PerCP-Cy5.5, CD45RO-FITC, CD3-V500, CD4-V450, CD27-APC (all from BD Biosciences).

### Western blot

Cells were serum-starved for 4 h, incubated with anti-CCR7 mAb (10 μg/mL) or the appropriate IC, treated with hCCL19 or hCCL21 (1 μg/mL, PeproTech, Rocky Hills, NJ) for the indicated times and then lysed in ice-cold modified-RIPA buffer. Equal amounts of protein were analyzed by SDS-PAGE and immunoblotting using phospho-ERK1/2 (Thr202/Tyr204), phospho-AKT (Ser473), phospho-MLC (Ser-19), anti-AKT, anti-ERK1/2 and anti-MLC specific antibodies, all of them purchased from Cell Signaling Technology (Danvers, MA). Membranes were first probed with antibodies against the phospho-proteins under study, and then re-probed with antibodies recognizing the total amount of each protein. Bands were visualized using enhanced chemiluminescence (Amersham Pharmacia Biotech, Buckinghamshire UK), and quantified with a LAS1000 image analyzer (FujiFilm, Tokyo, Japan).

### Migration

Chemotaxis of T-PLL cells was assayed in Transwell chambers (6.5 mm diameter, 10 μm thickness and 5 μm diameter pore size, Corning-Costar, Tewksbury, MA) as previously described [23]. Only samples from patients with > 95% of tumor cells were analyzed. Briefly, 5 × 10^5^ cells suspended in RPMI-1640, 0.5% BSA were loaded in the upper chamber and chemokines (1 μg/mL) were added to the lower compartment. Migration was allowed to proceed for 4 h at 37 °C and 5% CO_2_. When required, cells were pre-incubated for 30 min with anti-CCR7 mAb (10 μg/mL) or an IC, which were maintained throughout the chemotaxis assays. Cells that migrated to the lower chamber were stained with anti-CD3-PE mAb (clone SK7, BD Biosciences) and anti-CD7-APC (clone M-7 T01, BD Biosciences), enumerated by flow cytometry using a FACS Canto II flow cytometer and compared with the number of cells loaded in the upper chamber. The percentage of migrated cells (% of input) was calculated according to the following formula: 100 x (number of cells in the lower chamber / number of cells loaded in the upper chamber).

### Invasion

Three million serum-starved T-PLL cells were harvested and included in 4 °C liquid Matrigel™ (BD Biosciences Pharmingen, San Diego, CA). Then, the Matrigel suspension containing cells was added to the Transwell chambers (Costar) and incubated at 37 °C for 30 min. Cells were incubated with anti-CCR7 mAb (10 μg/mL) or IC prior to addition of Matrigel. Once Matrigel was solidified the chemokines were added to the lower chamber and cells were allowed to migrate in RPMI-1640 with 1% FBS for 24 h at 37 °C in 5% CO_2_ atmosphere. Cells that had migrated to the lower chamber were quantified as described in migration assays.

### Transendothelial migration (TEM)

Tumor cell transmigration through intact endothelium was examined using monolayers of cultured human umbilical vein endothelial cells (HUVEC) seeded in gelatine-coated Transwell chambers (Costar) at a density of 4 × 10^4^ cells per well as previously reported [24]. The integrity of HUVEC monolayers was confirmed by toluidin blue staining and confluent monolayers were stimulated with 15 ng/mL recombinant TNF-α (R&D Systems), for 16 h prior to the assay. Before applying to upper chambers, T-PLL cells were incubated with anti-CCR7 mAb (10 μg/mL) or an IC. Then, leukemic cells (5 × 10^5^ per well) were suspended in medium with 1% FBS, and placed into upper chambers. Lower chambers were filled with medium containing hCCL19 or hCCl21 (1 μg/mL). After 4 h, cells in the lower chamber were recovered, stained and enumerated as described above.

### Gelatin zymography

T-PLL cells (3 × 10^6^) were placed in serum-free media for 1 h and then treated with either IC or anti-CCR7 mAb (10 μg/mL) for 30 min. Cells were left untreated or stimulated with CCL19 or CCL21 (1 μg/mL) for 24 h. Conditioned media were collected and centrifuged to remove cells or debris. Samples were concentrated using a Centricon (Millipore, Burlington, MA) to identical final volumes for all conditions. Zymography was performed on a 10% SDS-polyacrylamide gel incorporated with 0.1% gelatin (Sigma, San Luis, MO) [24]. FBS, containing MMP-2 and MMP-9, was used as a positive control. After electrophoresis, MMPs were allowed to re-nature by washing the gel twice in 50 mL of 2.5% Triton X-100 for 30 min, incubated for 24 h at 37 °C in developing buffer (50 mM Tris pH 7.5, 200 mM NaCl, 10 mM CaCl_2_), and stained for 1 h with 0.5% Coomassie brilliant blue G250. After de-staining, gelatinolytic activity was visualized as a transparent band against a blue background. Analysis of proteolytic bands was performed using LAS1000 image analyzer (FujiFilm).

### Proliferation

T-PLL cells (5 × 10^5^) were stained with 5 mM CellTrace™ Violet reagent (Molecular Probes, Eugene, OR) prior to stimulation with CCL19 and CCL21 (1 μg/mL). Cell labelling was performed according to the manufacturer’s protocol. Cells suspended in 1% FBS RPMI-1640 complete medium were then seeded in 96-well plates, stimulated with chemokines and incubated for 7 days. Every 24 h a total of 5 × 10^4^ cells were analyzed on a FACS CantoII BD with 405 nm excitation and a 450/40 band-pass emission filter in order to discriminate discrete peaks representing successive generations of live T-PLL cells. The growth rate was calculated according to the formula: GR = [CellTrace™-positive-cells(t0) – CellTrace™ -positive-cells (tX)] / CellTrace™ -positive-cells(t0).

### Cell survival

To examine whether CCR7 is involved in survival of T-PLL, leukemic cells suspended in 1% FBS RPMI-1640 complete medium were seeded in 96-well plates at a density of 10^6^ cells/mL. Cells were either left untreated or incubated for 30 min in the presence of anti-CCR7 mAb (10 μg/mL) before the addition of the ligands (1 μg /mL). Cell viability was determined every 24 h by staining with the Annexin-V and the DNA dye 7-aminoactinomycin-D (7-AAD) kit from BD Biosciences. The apoptosis assay was used according to the manufacturer’s instructions to determine the percentage of non-viable cells by flow cytometry.

### Apoptosis

To assess whether anti-CCR7 mAb induces direct apoptosis, T-PLL cells were incubated with anti-CCR7 mAb in RPMI-1640 complete medium. The cells were incubated at 37 °C for a maximum of 72 h. Every 24 h cells were stained with the Annexin-V/7-AAD assay as described before. Then we carried out the quantitative determination of the percentage of viable and non-viable cells.

### Complement-dependent cytotoxicity (CDC)

CDC assays were performed as previously described [25, 26]. Briefly, 2 × 10^5^ T-PLL target cells were plated in a 96-well round-bottom plate together with the indicated concentrations of purified anti-CCR7, alemtuzumab (anti-CD52; Campath™, Genzyme, Cambridge, MA) or IC antibodies. After 30 min at 37 °C the cells were washed and complete RPMI-1640 medium containing 25% rabbit complement (Serotec-Bio-Rad, Hercules, CA), with or without prior heat inactivation (56 °C, 30 min), was added. After 1.5 h, 7-AAD was used as a viability exclusion dye and the percentage of non-viable cells was measured by flow cytometry. The proportion of specific lysis (% SL) was calculated with the formula: % SL = 100 x (% dead cells with activated complement – % dead cells with inactivated complement) / (100 – % dead cells with inactivated complement).

### Antibody-dependent cell-mediated cytotoxicity (ADCC)

ADCC assays were performed as previously described [25, 26]. Cells were incubated with media alone or in the presence of IC, alemtuzumab, or anti-CCR7 antibodies (10 μg/mL) at 37 °C for 30 min. Unbound antibody was washed off and the cells plated at 10^4^ cells/well. Isolated NK cells from healthy donors or wild-type mice were labeled with calcein-UV Cell Tracker (Invitrogen, Carlsbad, CA) and stimulated with IL-2 (R&D Systems). NK cells were used as effector cells at effector: target ratios (E:T) of 6:1. After 4 h, cells were stained with 7AAD, and analyzed by flow cytometry. The percentage of specific lysis was determined by: %SL = 100 × (ER-SR)/ (MR-SR). ER, SR, and MR represent experimental, spontaneous and maximum cell death. Data were normalized to the media control.

### In vivo studies

In vivo procedures were carried out at CBM-SO (Madrid, Spain) in accordance with the guidelines approved by the Animal Experimentation Ethics Committee of the Spanish National Research Council. For the in vivo proof-of-concept analyses, a novel systemic xenograft model was developed by engrafting the human t (14;14)-carrying T-cell leukemia line SUP-T11 into sub-lethally irradiated (1.5 Gγ) immunodeficient mice. To this end, 5 × 10^5^ cells/mouse were intravenously (*i.v.*) injected into 6–10 weeks-old RAG2^−/−^γc^−/−^ [27] or NOD.Cγ-Prkdcscid-IL2rgtm1Wjl/SzJ mice (NSG; The Jackson Laboratory, Bar-Harbor, ME). Bioluminescent models were performed by transducing SUP-T11 cells with lentiviruses [28]. To measure luminescence, 150 mg/Kg of D-Luciferin was administrated intraperitoneally and tumor burden was monitored using the Xenogen IVIS Lumina II imaging system (Caliper Life Sciences, Waltham, MA). The photon flux emitted by the luciferase-expressing cells is measured as an Average radiance (photons/sec/cm^2^/sr). Imaging analysis is performed using the Living Image™ Software 3.2 (Caliper Life Sciences). Mice showing local tumor growth at the injection site (and which developed no systemic disease therefore) were discarded from analyses and sacrificed for ethical reasons. Similarly, enrolled animals showing endpoint weight loss or clinical signs were euthanized. PB and organs [spleen, lymph node (LN), bone marrow (BM), brain, lungs, and liver] were harvested in every animal. From each organ collected, one half was mechanically disaggregated and conserved in PBS at 4 °C for flow cytometry analyses. The percentage of tumor cells was determined using specific (non cross-reacting with murine cells) anti-hCD45-PerCP (clone 2D1) and anti-hCD5-APC (clone L17F12) mAbs (all from BD Biosciences). In some cases, the viability of tumor cells was quantitatively determined by flow cytometry with the Annexin-V-FITC/7-AAD assay on SUP-T11 cells. Similarly, proliferation was determined by staining with anti-Ki67-PE (BD Pharmingen). The distribution of leukemic cells within the different organs was determined by immunohistochemistry (IHC) against hCD45. Briefly, halves of organs and tissues extracted from mice were fixed in 4% neutral buffered formaldehyde, paraffin-embedded, and sliced in 4 μm sections. For human CD45 inmmunohistochemistry, the antigen retrieval was performed with tris-EDTA pH 9. Slides were stained with 1:100 anti-hCD45 (clone 2B11&PD7/26, Cell Marque, Rocklin, CA) by using the Dako REAL EnVision Detection System (Dako, Glostrup, Denmark).

### Statistics

Unless otherwise stated, quantitative variables are expressed as measures of central tendency (mean) and dispersion (SD, SEM). Quantitative variables with equal variances (Levene’s test) were analyzed using t-test or ANOVA. Mann-Whitney-U or Kruskal-Wallis tests were used for heteroscedasticity. Survival curves were analyzed by the Kaplan-Meier method and log-rank test. A Cox regression model was used to estimate cumulative risk for OS (hazard ratio, HR) vs proportion of CCR7^+^ T-PLL cells. Significance was set at values of < 0.05(*), < 0.01(**) or < 0.001(***).

## Results

### CCR7 is a functional receptor highly expressed on T-PLL cells

**Fig. 1:**
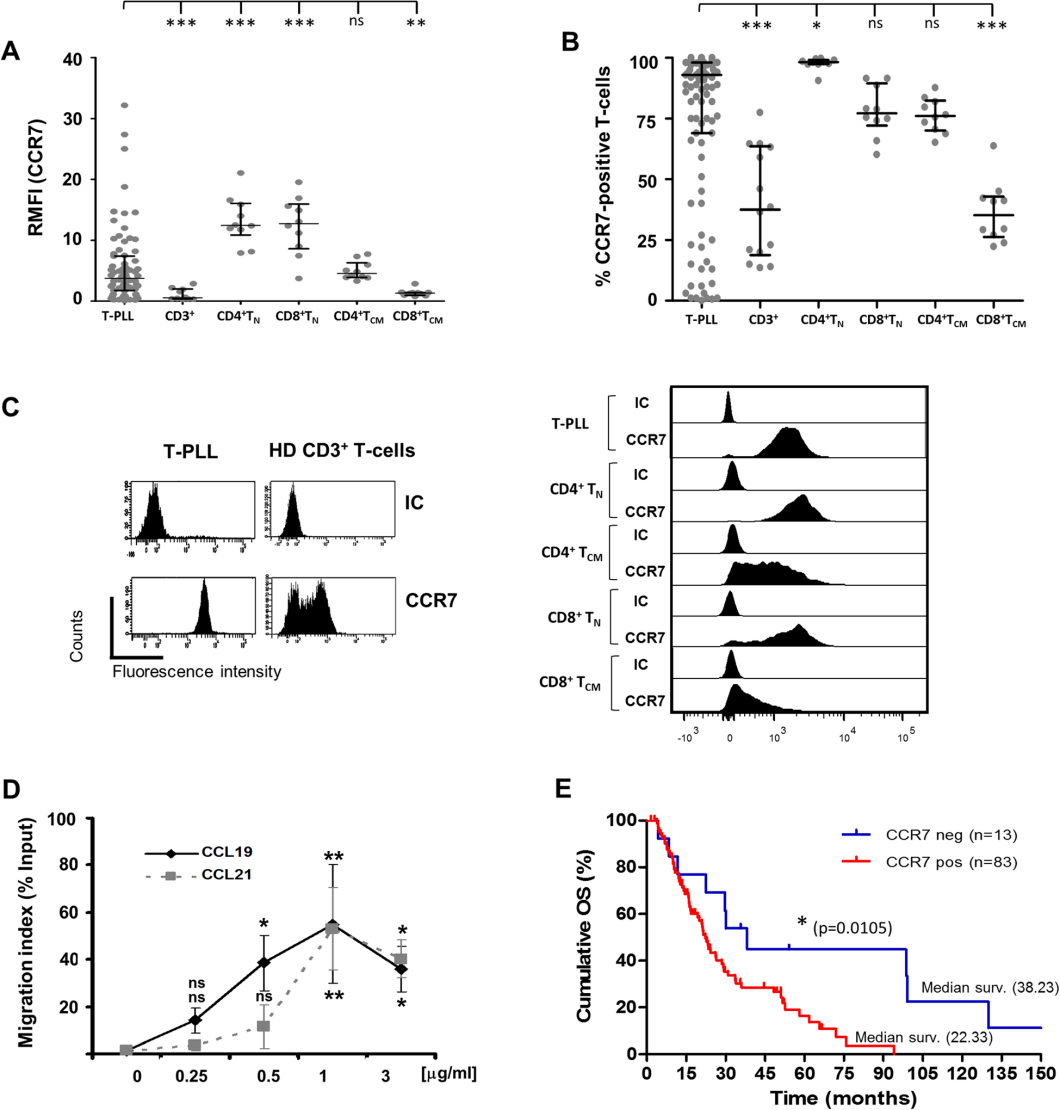
T-PLL cells express high levels of functional CCR7 that is associated with shorter overall survival (OS). **a** CCR7 relative median fluorescence intensity (RMFI) in T-PLL cells compared to T-cells from healthy donors. T-cells obtained from PB samples of patients (*n* = 75) or healthy donors (*n* = 10) were analyzed by flow cytometry to determine CCR7 surface density measured as MFI relative to a corresponding IC. **b** Proportion of CCR7-positive T-cells is high in T-PLL. The percentage of CCR7-positive T-cells was determined in T-PLL patients (*n* = 109) and healthy donors (*n* = 14). In A-B the median ± interquartile range is shown. **c** CCR7 expression in pan T-cells or specific CCR7-expressing T-cell subsets (T_N_, T_CM_) from a representative patient and a healthy donor. The expression pattern and intensity of CCR7 is shown. **d** T-PLL cells migrate towards CCR7 ligands. Chemotaxis assays with T-PLL cells (*n* = 5) were performed with different doses of CCL19 and CCL21 (x axis). Each symbol represents the percentage of migrated cells in each experimental condition relative to the number of cells loaded in the upper chamber (% input). The mean ± SEM is shown. **e** CCR7 surface expression on T-PLL cells is associated with OS. The surface expression (% positive cells) of CCR7 was assessed by flow cytometry in T-PLL patients with clinical follow-up data (*n* = 96). At diagnosis, cases were classified as CCR7 positive (> 20% CCR7^+^ T-PLL cells) or negative (< 20%). OS was estimated and compared by Kaplan–Meier curves and log-rank test, respectively. The p-value of the log-rank statistical test is given. *, *p* < 0.05; **, *p* < 0.01; ***, *p* < 0.001; ns, not significant

B-cell malignancies with high levels of CCR7 show a widespread nodular dissemination [29]. As T-PLL displays such an infiltration pattern, we asked whether CCR7 levels were likewise augmented in this disease. CCR7 surface expression was analyzed, at diagnosis, by flow cytometry on malignant T-cells in peripheral blood (PB) from 109 T-PLL patients, and on T-cells from 14 healthy donors. We found that CCR7 expression in T-PLL lymphocytes was consistently higher than in normal PB pan-T-cells [median of fluorescence intensity relative to the control (RMFI) in T-PLL vs healthy T-cells: 5.591 ± 0.7163 vs 1.094 ± 0.3021; Fig. 1 a]. Since surface chemokine receptor expression is sensitive to several factor, we ruled out that CCR7 expression was not affected by antibody-induced internalization (Supplementary Figure 1**)**, the anticoagulant used, time from sample extraction to flow cytometry tests, or isolation processes in fresh samples at diagnosis (data not shown). Also, a higher proportion of CCR7-expressing cells was found in T-PLL samples (T-PLL vs healthy T-cells: 75.95 ± 3.187 vs 39.76 ± 6.030, Fig. 1 b), where CCR7 expression was found to be homogeneous whereas a bimodal distribution was seen in healthy PB pan-T-cells due to the presence of CCR7-positive (T_N_, T_CM_) and CCR7-negative subsets (effector cells) in a given sample (Fig. 1c). To further characterize CCR7 expression we also compared surface levels in our T-PLL cohort to the expression in different non-tumor T-cell subsets including CCR7-expressing T_N_ and T_CM_ cells (in both CD3^+^CD4^+^ and CD3^+^CD8^+^ subsets). Results shown in Fig. 1a,b,c reveal that there is no overall difference in RMFI or in the proportion of CCR7-expressing cells between T-PLL and normal CD4^+^ T_CM_ cells. In addition, a remarkable proportion of T-PLL cases showed a CCR7 profile similar to a T_N_ cell profile. These results suggest that CCR7 expression in T-PLL comprises a spectrum of differentiation ranging from T_N_ to T_CM_ cell phenotypes. Accordingly, when we studied the flow-cytometry based expression pattern of CD45RO/CD45RA in our cohort, we could corroborate that most cases resembled a memory phenotype and that similar proportions of memory subsets were found in CCR7-negative and CCR7-positive groups (Table 1; RA^+^RO^+^: 33% in CCR7^−^ vs 25% in CCR7^+^; RA^−^RO^+^: 66% vs 67%).

**Table 1:** CD45RO/RA pattern of CCR7-negative vs CCR7-positive T-PLL cases (*n* = 82 cases)

	CCR7^-^ (number of cases)	CCR7^**+**^ (number of cases)
**Naïve CD45RO**^**−**^**CD45RA**^**+**^	0	3
**Pan-memory CD45RO**^**−**^**CD45RA**^**−**^	0	2
**Transition CD45RA**^**+**^**/RO**^**+**^	3	16
**Central or effector memory CD45RA**^**−**^**/RO**^**+**^	6	43
**Median age and range (years)**	68 [32–81]	
	Fisher’s exact test (*p* = 0.86) Pearson’s Chi-squared test (*p* = 0.82).	

Finally, CCR7 was functional in T-PLL cells and triggered migration towards its cognate ligands, the chemokines CCL19 and CCL21. CCR7 induced a bell-shape dose-dependent migratory response with a maximum induction by 1 μg/mL with both chemokines (Fig. 1d), a concentration within the range estimated to exist in T-zones of lymph nodes (LN) [30].

### CCR7 expression is associated with shorter overall survival of T-PLL patients

CCR7 expression on malignant cells is associated with poor OS in solid tumors [31]. Nonetheless, such correlation has not been described before in blood cancers. To address this question, we dichotomized the uniformly treated T-PLL patients (backbone of alemtuzumab induction) of our cohort by setting a cut-off equal to the 20.5% of CCR7^+^ T-PLL cells as determined by ROC analysis (sensitivity of 84.55%; 95% CI: 76.93 to 90.44%). This cut-off agreed with those of various cell makers previously described [32].

Patients were then grouped into CCR7-positive [> 20% positive cells; 83/96 (86.5%) patients] or CCR7-negative [≤20%; 13/96 (13.5%)]. Accordingly, a density plot showing the distribution of CCR7 positivity across all cases clearly indicated that CCR7 expression was divided into these two groups (Supplementary Figure 2) and precluded the use of a continuous expression of CCR7 in survival analyses. With these settings, the median OS period was higher in CCR7-negative patients (Fig. 1e: 38.23 vs 22,33 months; log-rank *p* = 0.0105; HR = 0.4612; C.

I (95%) = 0.2549–0.8346). Four years after diagnosis, 44,8% of CCR7^−^ patients survived whereas this proportion decreased in the CCR7^+^ group to 26.65%; eight years after diagnosis the proportions were 33% in CCR7^−^ patients vs 0% in CCR7^+^ patients. Fifteen cases in the CCR7^+^ group and 4 in the CCR7^−^ subset received an allo-HSCT. To further study causality between expression of CCR7 and OS, Cox regression analyses were conducted (Table 2). No association with OS was found when we tested a continuous expression of CCR7, however, a HR of 2.426 (*p* = 0.044) was found for cases expressing CCR7 as compared to negative cases. Notably, when risk analyses took into account only patients with positivity for CCR7 (Table 2) no significant differences in risk were found, indicating that all patients with expression of CCR7 had similar poor outcomes regardless of the proportion of CCR7^+^ T-PLL cells at diagnosis.

**Table 2:** Cox regression analyses

	HR	***p***-value	95% CI	n° cases	LR Chi2	Prob > Chi2
**% CCR7** **(continuous)**	1.004	0.235	0.997–1.013	92	1.52	0.217
**% CCR7** **(high/low)**	2.426	**0.044**	1.026–5.739	93	5.08	0.024
**% CCR7 within the CCR7-high group (continuous)**	0.988	0.092	0.973–1.002	80	2.43	0.119

*Abbreviations*: *CI* Confidence interval, *HR* Hazard ratio, *LR* Likelihood ratio; high: ≥20% positive T-PLL cells by flow cytometry; low: < 20% positive T-PLL cells by flow cytometry

### CCR7 ligands trigger receptor signaling, a process effectively blocked by CCR7 mAbs

The expression and functionality of CCR7 in T-PLL suggested this molecule to be interrogated as a therapeutic target in T-PLL. Given the lack of knowledge on the role of chemokines receptors in T-PLL, we studied in more detail the operational CCR7-downstream pathways and the mechanisms of action (MOA) mediated by a blocking anti-CCR7 mAb on primary T-PLL cells. This characterization included analyses of the activity displayed by the Fab region (which can block the target/ligand interaction) and by the Fc region, which can engage the immune cells or proteins, thus promoting effector activities.

**Fig. 2:**
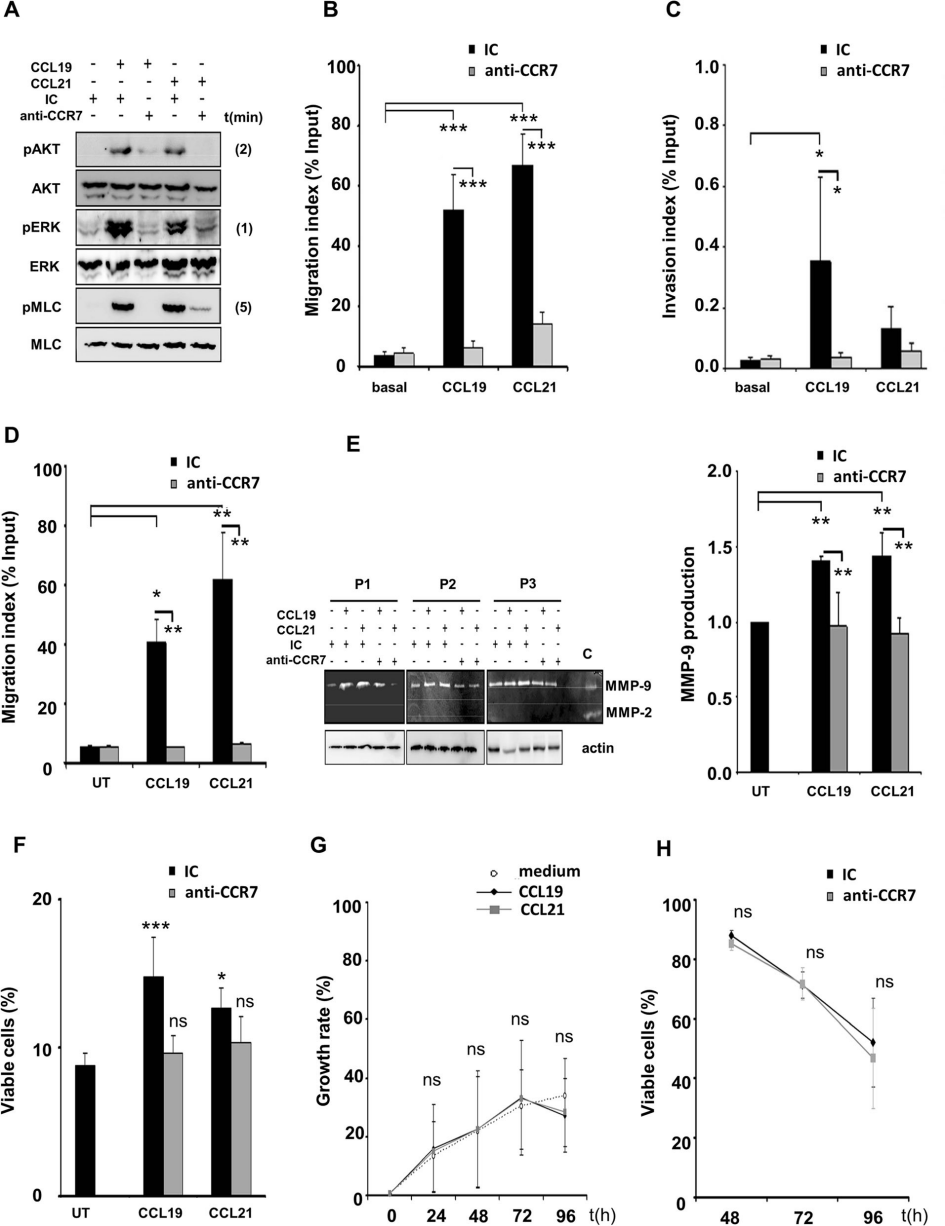
Blocking CCR7 neutralizes target-mediated cell functions on T-PLL. **a** Anti-CCR7 mAb blocks CCR7 activation and signal transduction in T-PLL cells. Serum-starved T-PLL cells were pre-treated with 10 μg/mL of anti-CCR7 or the respective IC for 30 min and then treated with CCL19 or CCL21 for different time points. Cell extracts were analyzed by Western blot. The figure shows representative blots from 6 independent experiments. **b** Anti-CCR7 mAb neutralizes CCR7-mediated T-PLL cells migration in response to CCL19 and CCL21. Chemotaxis of T-PLL cells induced by CCL19 and CCL21 was assayed in uncoated transwell chambers for 4 h. When indicated, cells were pre-treated with 10 μg/mL of anti-CCR7 mAb or the respective IC (*n* = 6). **c** Blocking CCR7 abrogates T-PLL cells invasion driven by CCL19 and CCL21. T-PLL cells with or without previous incubation with anti-CCR7 mAb or the matching IC were embedded in Matrigel™ before exposure to chemokines for 24 h. Values represent the percentage of migrated cells referred to the total cells added (*n* = 4). **d** Anti-CCR7 mAb inhibits target-mediated T-PLL cells migration across endothelium. T-PLL cells were incubated on the upper chamber of transwell filters coated with TNFα-activated HUVEC, in the presence or absence of the indicated mAbs. Then chemokines were added and after 4 h, the cells were counted by flow cytometry. Values represent the percentage of migrated cells referred to the total cells added (*n* = 4). **e** Anti-CCR7 mAb reduces target-mediated MMP-9 secretion. CCR7 engagement up-regulates MMP-9 in T-PLL cells. Leukemic cells from 3 different patients (P1, P2 and P3) were pre-treated with anti-CCR7 mAb or an IC (10 μg/mL) and then incubated with/without CCL19 or CCL21 (1 μg/mL) for 24 h. The concentrated conditioned media were analyzed by gelatin zymography to detect MMP-2 and MMP-9 secretion. MMP-9 was identified as the 92-kDa proactive form. Cell pellets of each point were lysed and cell extracts were used to detect actin by Western blotting as loading control (C, fetal bovine serum (FBS) used as positive control showing the degradation bands of active MMP-9 and MMP2). The average quantification of MMP-9 secretion (arbitrary units) for the 3 samples studied is given; basal levels of MMP-9 on bovine serum albumin (BSA) and without chemokines were normalized to 1. **f** Anti-CCR7 mAb affects target-induced pro-survival signaling pathways. CCR7 ligands increase T-PLL cells survival, a process blocked by anti-CCR7 mAb. Cells from 4 patients were incubated in 1% FBS medium alone or with CCR7 ligands (1 μg/mL) for 72 h. Where indicated, cells were also incubated in the presence of 10 μg/mL of anti-CCR7 mAb or an IC. The percentage of viable 7-AAD^−^ Annexin-V^−^ cells at 72 h is shown. **g** CCR7 ligands do not induce proliferation of T-PLL cells. T-PLL cells (*n* = 5) were harvested and stained with CellTrace™ Violet reagent. Then, 5 × 10^5^ cells were cultured with 1% FBS complete medium alone or supplemented with CCL19 or CCL21. Every 24 h the successive generations of live cells were analyzed. **h** Anti-CCR7 mAb does not induce direct apoptosis on T-PLL cells. Leukemic cells were incubated with anti-CCR7 (10 μg/ml) or the corresponding IC in complete medium with 10% FBS (*n* = 5). Every 24 h the effect of the antibody was studied by Annexin-V/7-AAD double staining. In B, C, D, E, F, G, H, bars (or symbols) represent mean ± SEM. *, *p* < 0.05; **, *p* < 0.01; ***, *p* < 0.001; ns, not significant

First, we explored MEK/ERK1/2, PI3K/AKT, and Rho/ROCK/MLC as the main CCR7 signaling cascades [23] to be inducible in primary T-PLL cells upon ligand binding. In 6 samples tested, both ligands induced ERK1/2, AKT, and MLC phosphorylation, which peaked after 1, 2, and 5 min, respectively (Fig. 2 a). Additionally, we confirmed that activation of these cascades was blocked by an anti-CCR7 mAb.

### CCR7 mediates T-PLL cell migration and invasion, which are neutralized by CCR7 mAbs

As CCR7 mediated T-PLL cell chemotaxis (Fig. 1d), we postulated that neutralizing this process could prevent leukemic dissemination to CCL19/CCL21 producing locations (SLO and CNS). As a proof-of-concept, anti-CCR7 mAb blocked in vitro migration of primary T-PLL cells towards CCL19/CCL21 in uncoated transwell chambers (Fig. 2b). The mAb also abrogated cell processes necessary for the migratory response [33] such as actin-skeleton reorganization (Supplementary Figure 3-A-B) or the development of uropods (Supplementary Figure 3-C-D). Furthermore, we performed chemokine-induced invasion assays in transwell chambers coated with Matrigel (Fig. 2c). Compared to basal invasion, both ligands increased the number of invading cells (albeit CCL19-mediated invasion seemed stronger) and such invasion was almost completely inhibited by the anti-CCR7 mAb.

Through TEM assays we also confirmed that T-PLL cells efficiently migrated across endothelium (HUVEC monolayers) in response to CCL19/CCL21 (Fig. 2d). In these experiments, CCL21 outperformed CCL19 probably due to the ability of CCL21 to bind endothelium through its C-terminal tail [34]. Again, the anti-CCR7 mAb completely blocked migration towards both chemokines (Fig. 2d). Release of the gelatinases MMP-2 and -9 by tumor cells degrades matrices and/or basement membrane, which is a key step in invasion/metastasis. To assess whether these enzymes are regulated by CCR7 ligation, T-PLL cells were incubated with/without chemokines and cell media were then analyzed by gelatin zymography. Figure 2e shows that tumor cells constitutively secreted activated MMP-9 with no traces of activated MMP-2. Although CCL19/CCL21 promoted differential invasion across Matrigel™ or HUVEC (Fig. 2c.d), both chemokines significantly increased the secretion of MMP-9 in zymographies to a similar extent, which was in turn blocked by exposure to anti-CCR7 mAbs (Fig. 2 e).

### T-PLL cell survival, promoted by CCR7 ligands, is impaired by anti-CCR7 mAbs

CCR7 promotes cell survival and proliferation in different hematological malignancies [23, 35]. Here, exposure of T-PLL cells to either CCL19 or CCL21 induced a moderate but significant increase of viability in long-term suspension cultures (Fig. 2f) without affecting proliferation (Fig. 2 g). This proactive impact on cell viability was abrogated by the anti-CCR7 mAb, which likely promoted cell death by blocking these CCR7-mediated pro-survival signals because this antibody did not mediate direct cell death upon target binding in the absence of these chemokines (Fig. 2h).

### Anti-CCR7 mAb triggers a strong CDC activity against T-PLL cells

**Fig. 3:**
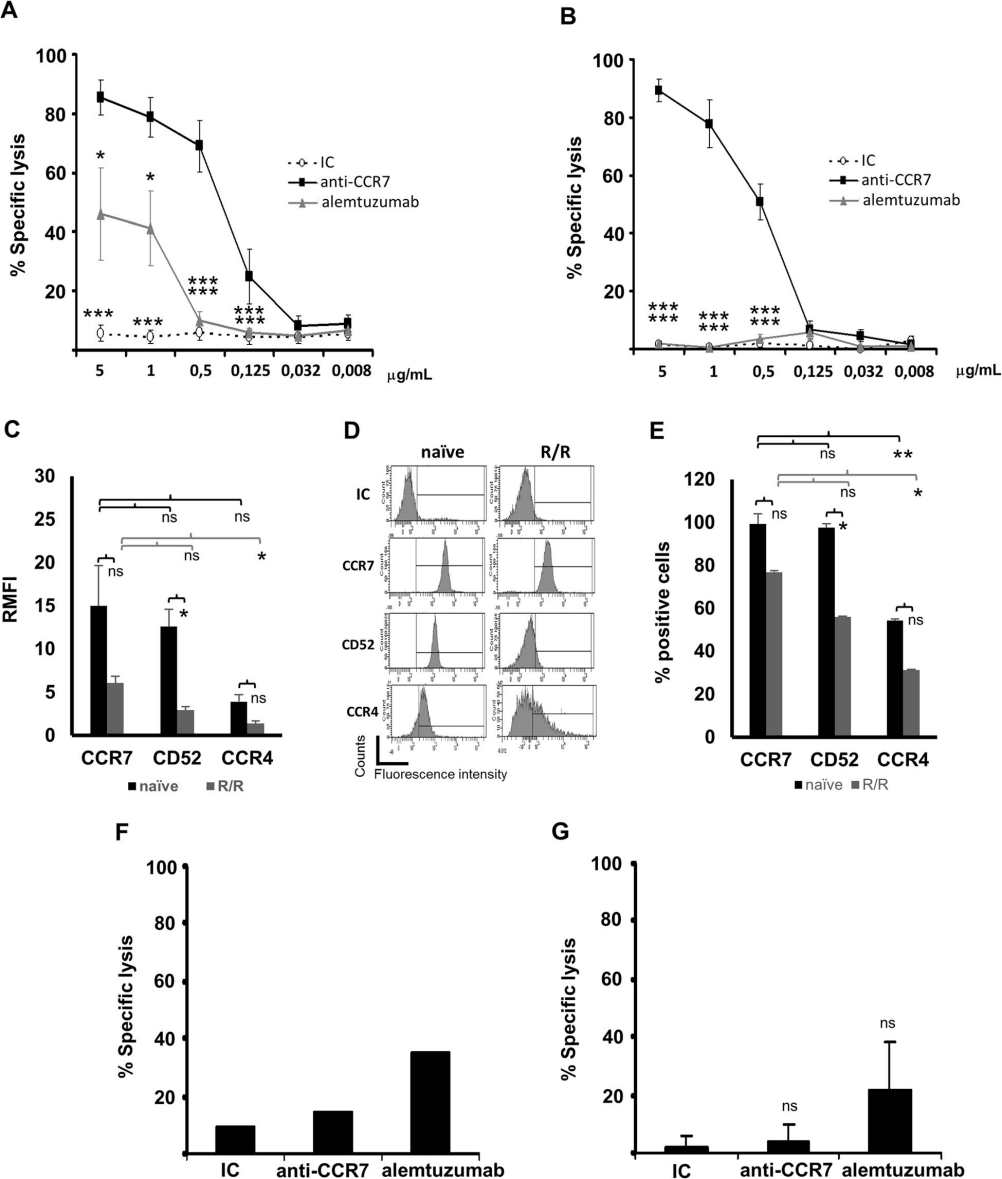
Anti-CCR7 mAb mediates a strong CDC on T-PLL cells. **a** CDC on alemtuzumab-naïve patients. Leukemic cells from T-PLL patients (*n* = 8) were incubated with anti-CCR7, anti-CD52 (alemtuzumab), or an IC at the indicated concentrations and then exposed to rabbit complement for 1.5 h. The percentage of specific cell lysis was determined through quantification of 7-AAD incorporation by flow cytometry. **b** CDC on alemtuzumab-refractory patients. Specific lysis on T-PLL cells from 4 refractory patients was assayed as described in A. **c** Relative median fluorescence intensity (RMFI) of CCR7, CD52 and CCR4 in T-PLL cells from alemtuzumab-naïve or alemtuzumab-refractory patients (R/R). Cells obtained from PB samples of patients not treated with alemtuzumab (n = 8; black bars) or refractory to this mAb (*n* = 4; grey bars) were analyzed by flow cytometry to determine CCR7, CD52, and CCR4 surface density measured as MFI relative to a corresponding IC. d CCR7, CD52, and CCR4 surface expression in T-PLL cells from one representative alemtuzumab-naïve or -refractory patient. E) Proportion of CCR7^+^, CD52^+^ and CCR4^+^ in T-PLL cells from alemtuzumab-naïve or alemtuzumab–refractory patients. The percentage of positive cells was determined in naïve (n = 8; black bars) and refractory patients (n = 4; grey bars). **f** and **g** Anti-CCR7 antibody does not mediate antibody-dependent cell-mediated cytotoxicity (ADCC). T-PLL cells were incubated with media alone or in the presence of an IC, anti-CD52 (alemtuzumab) or anti-CCR7 antibodies. **f** Isolated and IL-2-stimulated human NK cells from a healthy donor were used as effector cells at an effector to target (E:T) ratio of 6:1 (*n* = 1). **g** Isolated and IL-2-stimulated wild-type mouse NK cells from spleen (*n* = 2) were used as effector cells at an effector to target (E:T) ratio of 6:1. After 4 h, the percentage of T-PLL cells killed by ADCC was determined through 7-AAD staining by flow cytometry. In A, B, C, E, F, and G, bars (or symbols) represent mean ± SEM. *, *p* < 0.05; **, *p* < 0.01; ***, *p* < 0.001; ns, not significant

Therapeutic mAbs can induce target cell death through Fc-mediated cytotoxicity, including CDC and ADCC. In this study, the anti-CCR7 mAb triggered a strong in vitro CDC activity against T-PLL cells obtained from 8 patients not treated before with alemtuzumab (Fig. 3 a). This was observed at concentrations as low as 0.125 μg/mL. Similarly, alemtuzumab mediated a significant CDC but was outperformed by anti-CCR7 mAb at dose ranges of 0.125–5 μg/mL. Anti-CCR7 exposure was also highly effective against cells from four alemtuzumab-relapsed/refractory (R/R) patients (Fig. 3 b) and against cells from SS patients (Supplementary Figure 4).

The differences in CDC observed between alemtuzumab and the anti-CCR7 mAb can be attributed to a different isotype (human IgG1 vs mouse IgG2a), but also to different target surface expression levels. Compared with CD52, which is highly expressed on T-prolymphocytes [36], CCR7 levels were slightly higher in T-PLL cells from treatment naïve patients (Fig. 3 c,d,e). Accordingly, significantly diminished levels of CD52 where seen in three out of four R/R patients while the other lacked the target (Fig. 3 c,d,e). In R/R patients a non-significant reduction of CCR7 expression was also observed. Despite this, CCR7 expression levels were enough to mediate CDC upon anti-CCR7 treatment, while alemtuzumab-mediated CDC was abolished (Fig. 3b).

Another receptor targeted in T-cell malignancies is CCR4. In both naïve and alemtuzumab-R/R patients, expression of CCR4 was much lower than CD52 and CCR7 (Fig. 3 c,d,e) thus suggesting that anti-CCR4 antibodies, such as mogamulizumab, are not suitable for treating T-PLL. As seen with CCR7 and CD52, CCR4 expression was also decreased in R/R patients, thus indicating that down-regulation of surface markers is a common event in such population.

Finally, anti-CCR7 mAb did not trigger ADCC of T-PLL cells mediated by the recruitment of effector mononuclear myeloid cells (data not shown) or NK cells isolated from human PB (Fig. 3f) or from mouse spleens (Fig. 3 g), suggesting that anti-CCR7 mAb was ineffective in activating the FcγR on immune cells.

### Antibody-based CCR7 targeting is effective in novel xenograft models of T-PLL

**Fig. 4:**
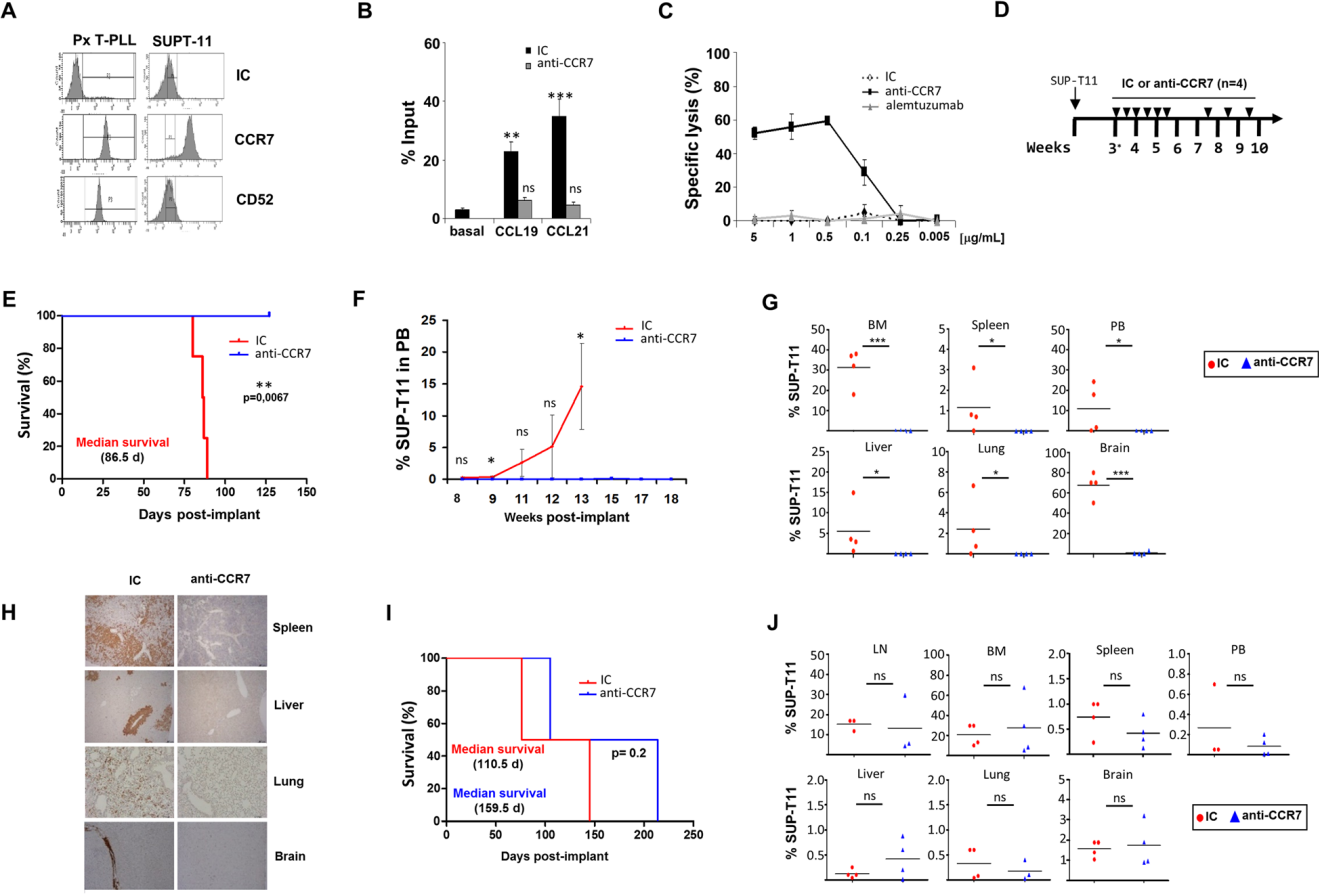
Anti-CCR7 is effective as a monotherapy in a novel post-implantation disease model of T-PLL. **a** SUP-T11 cells show similar CCR7 expression pattern than T-PLL patients. Tumor cells from one representative patient (Px) and from SUP-T11 cell line were stained with anti-CCR7 mAb, anti-CD52 mAb and an irrelevant IC. The graph shows frequency histograms with the pattern and intensity of each surface marker. **b** Anti-CCR7 mAb blocks migration of SUP-T11 cells. Chemotaxis of SUP-T11 cells induced by CCL19 and CCL21 (1 μg/mL) was assayed in uncoated transwell chambers (*n* = 3). Where indicated, cells were pre-treated with 10 μg/mL of anti-CCR7 or the respective isotype control, IC. Bars represent mean ± SEM. **c** Anti-CCR7 mediates CDC on SUP-T11 cells. Cells were incubated with anti-CCR7, anti-CD52 (alemtuzumab) or an IC at the indicated concentrations and then exposed to rabbit complement for 1.5 h (n = 2). Each symbol represents mean ± SD. **d** Schematic representation of model development and treatment schedule. The novel T-PLL-like systemic model was developed by intravenous inoculation of 5 × 10^5^ SUP-T11 cells into RAG2^−/−^γc^−/−^ deficient mice. On day 21 (*), when SUP-T11 cells were detected in bone marrow (BM), animals were randomized (*n* = 4 animals/group). Mice were treated intraperitoneally with 10 mg/kg (~ 200 μg/mouse) of anti-CCR7 mAb or its respective IC twice in weeks 3, 4, 5 and once in weeks 7, 8, 9 (arrows indicate antibody treatment days).** e **Anti-CCR7 mAb therapy increases survival of SUP-T11-xenografted mice. Survival was estimated and compared by Kaplan-Meier survival curves and log-rank test, respectively. **f** Anti-CCR7 mAb therapy reduces tumor burden in PB. The percentage of SUP-T11 cells in PB was determined by flow cytometry all over the experiment. Each point represents mean ± SD. **g** and **h** Anti-CCR7 mAb therapy impairs infiltration of lymphoid and non-lymphoid organs. **g** At sacrifice, one million cells from spleen, BM, PB, lung, liver, and brain were harvested and incubated with anti-hCD5 and anti-hCD45 antibodies. The percentage of SUP-T11 cells observed in each tissue is shown. Each symbol represents one individual mouse. Horizontal bars represent mean percentage of SUP-T11 cells of each group. **h** Immunohistochemistry (IHC) analyses were performed in tissue sections from spleen, liver, brain, and lungs using anti-hCD45 mAb. Representative pictures from both a control mouse and an anti-CCR7 treated mouse are shown. **i** and **j** Anti-CCR7 Fab-mediated MOA are insufficient to achieve the maximal therapeutic response. The novel TPLL-like systemic model was developed by intravenous inoculation of 5 × 10^5^ SUP-T11 cells into irradiated NSG deficient mice (see picture **d**). On day 21, and mimicking model on RAG2^−/−^γc^−/−^, animals were randomized (n = 4 animals/group). Mice were treated intraperitoneally with 10 mg/kg (~ 200 μg/mouse) of anti-CCR7 mAb or its respective IC twice in weeks 3, 4, 5 and once in weeks 7, 8, 9 (arrows indicate antibody treatment days). **i** Fab-mediated MOA in anti-CCR7 therapy induce a non-significant increase in survival. **j** Fab-mediated MOA in anti-CCR7 therapy do not reduce tumor cell infiltration. At sacrifice, flow cytometry analysis was performed as disclosed in **g**. Each symbol represents one individual mouse. Horizontal bars represent mean percentage of SUP-T11 cells of each group. *, *p* < 0.05; **, *p* < 0.01; ***, *p* < 0.001; ns, not significant

To further evaluate the utility of anti-CCR7 therapy in T-PLL we conducted in vivo proof-of-concept tests. Since the mAb did not recognize murine CCR7, we performed these studies in novel systemic xenogeneic models developed in host immunodeficient mice by intravenous injection of the SUP-T11 T-cell leukemia line [37], which, at the molecular level, resembles T-PLL as close as possible [38]. In these models, the tumor spread through PB, lymphoid organs (LN, spleen, and BM) and non-lymphoid tissues (liver, lung, and brain) (Supplementary Figure 5). Despite the fact that some characteristic surface markers of T-PLL were absent in SUP-T11 cells (e.g. CD4, CD8, CD25 and CD52), SUP-T11 was selected based on laboratory findings demonstrating a close resemblance with T-PLL regarding CCR7 (Fig. 4 a) and based on the presence of the T-PLL signature lesion of t (14;14)(q11;q13.2), an alteration found in other mature T-cell leukemia [39, 40], but extremely rare in immature stages [1, 41]_._ Here, we confirmed this translocation (and the resulting TCL1 protein overexpression) and, in addition, the strong positivity for CD7 and CD26 (another characteristic markers of T-PLL) [1], and the post-thymic, mature TCRαβ^+^ naïve phenotype characterized by intense expression of CCR7 and CD45RA, and negativity for CD34, CD10, TdT, CD117, HLA-DR, and the cortical thymic marker CD1a (Supplementary Figure 6). Finally, we determined that SUP-T11 cells migrated towards CCR7 ligands in vitro (Fig. 4 b) and confirmed that the anti-CCR7 mAb was effective in blocking such migration and in inducing CDC on SUP-T11 cells (Fig. 4 b,c).

To evaluate the in vivo anti-tumor efficacy of the anti-CCR7 mAb, first we used as hosts the RAG2^−/−^γc^−/−^ strain, which lacks T, B, and functional NK cells, but has complement activity [42], thus allowing in vivo testing of the MOA characterized in vitro*.* To better represent a clinical scenario for an anti-CCR7 treatment, we used a post-implantation set-up in which tumor cells had migrated to their target organs and niches (Fig. 4d). Accordingly, treatment was initiated 3 weeks after intravenous inoculation of SUP-T11 cells, once they were found in the BM (data not shown). Then, two groups (*n* = 4) were treated with either 200 μg/mouse (~ 10 mg/kg) of anti-CCR7 mAb or IC twice on weeks 3–5 and once on weeks 7–9. In this model, anti-CCR7 mAb significantly increased OS (Fig. 4e). While the survival rate in the IC group was 0% (median survival of 86.5 days), all mice treated with the anti-CCR7 mAb remained alive at the time when the last mouse in the control group had to be euthanized. Animals treated with the anti-CCR7 mAb did not develop clinical signs, gained weight and survived up to 127 days, the time when the animals were sacrificed which was considered as a bona fide disease-free period. Accordingly, no presence of SUP-T11 cells was detected in PB (Fig. 4f) whereas PB infiltration of control animals increased over the time. At the time of sacrifice, no SUP-T11 cells were seen in lymphoid and non-lymphoid tissues from anti-CCR7 treated animals. Conversely, there was consistent infiltration of these tissues in the control group (Fig. 4 g,h).

### In vivo anti-tumor activity of targeting CCR7 relies on both fab- and fc-mediated action

The potential of the anti-CCR7 antibody was further studied in irradiated NSG mice that besides lacking T, B, and NK cells, also lack complement activation [42]. These settings were used to examine the therapeutic benefit of blocking CCR7 by the Fab region, excluding Fc-mediated cytotoxicities. Of note, the anti-CCR7 mAb did not trigger in vitro FcγR-mediated activities on effector cells (Fig. 3 f,g) and irradiation provided a complementary way to abrogate activation of myeloid subsets. In this NSG model, we repeated the treatment schedule used in RAG2^−/−^γc^−/−^ mice (Fig. 4d). In the model the median OS in controls was 110.5 days, compared to 160 days in anti-CCR7 treated mice, and 50% of anti-CCR7 treated mice were alive when the last control mouse was euthanized (Fig. 4 i). Nonetheless, log-rank tests showed no statistical differences between the survival curves of both groups, and anti-CCR7 therapy did not significantly influence the presence of tumor cells in the tissues (Fig. 4j). Together, these results indicate that in anti-CCR7 mAb-based therapy both Fc- and Fab-mediated activities are necessary to achieve the maximum therapeutic benefit in T-PLL.

### Preventive anti-CCR7 mAb treatment tends to delay tumor onset and to reduce tumor burden in the T-PLL model

**Fig. 5:**
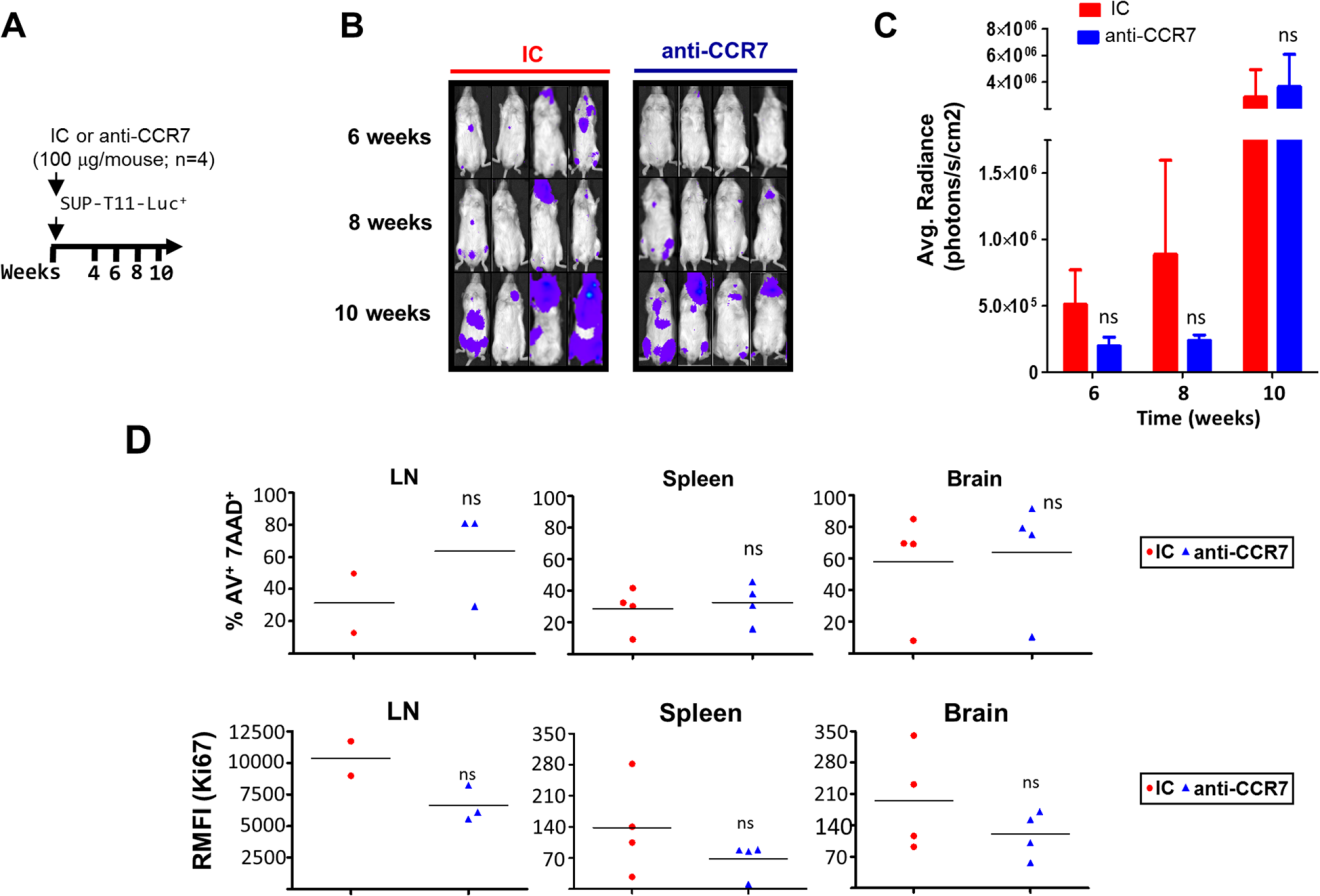
Anti-CCR7 Fab-mediated MOA moderately delay tumor onset and inhibit tumor burden in the SUP-T11-luc^+^ disseminated T-PLL models. **a** Schematic representation of development and treatment schedule in model 1. This preventive model was developed by treating irradiated NSG deficient mice with a single dose of 5 mg/kg (~ 100 μg/mouse) of either anti-CCR7 (*n* = 4) or the respective IC (*n* = 4) 2 h before the intravenous inoculation of 5 × 10^5^ SUP-T11-luc^+^ cells (arrow indicates antibody administration). **b-c** Anti-CCR7 mAb preventive therapy tends to reduce tumor burden and growth. Tumor burden follow-up was determined by whole body (back and front) bioluminescent analyses of each mouse on weeks 4, 6, 8, and 10. To simplify, only front images are shown (**b**). Quantification of luciferase activity is shown as mean ± SEM (**c**). **d** Blocking CCR7 seems to induce cell death in the LN and to reduce the proliferative phenotype in SUP-T11-luc^+^ cells migrated to the LN, spleen and brain. At sacrifice, one million cells from LN, spleen, and brain were harvested for flow cytometry analyses. Cells were incubated with anti-human-CD5 and anti-hCD45 antibodies, and with either Annexin-V/7-AAD, to quantitatively determine the proportion of non-viable cells, or with fluorochrome-conjugated antibody directed against the proliferative protein Ki67 in order to determine expression levels of this marker. The percentage of SUP-T11-luc^+^ dead cells and the median intensity of fluorescence of Ki67 relative to the IC (RMFI, arbitrary units) are shown. Each symbol represents one individual mouse. Horizontal bars represent the mean. ns, not significant

CCR7 plays critical roles in enabling tumor cells accessing and establishing tumor microenvironments (TME), e.g. shown in syngeneic models of Burkitt lymphoma [43] where genetic deletion of CCR7 delayed arrival at such milieus and niche formation, and impaired cross-talk between malignant and accessory cells [43]. Based on this, we aimed to study whether ‘preventive’ administration of anti-CCR7 mAb could impact disease establishment. To trace engraftment of tumor cells, we developed a new bioluminescent model in which SUP-T11 cells, stably expressing luciferase, were intravenously injected into NSG mice. Expression of CCR7 on these SUP-T11-luc^+^ cells was identical to their parental cells (data not shown). Two groups of 4 animals each were treated with either a single dose of 100 μg/mouse (~ 5 mg/kg) of the anti-CCR7 mAb or IC 2 h before intravenous inoculation of SUP-T11-luc^+^ cells (Fig. 5a). Bioluminescence images were obtained every 2 weeks until week 10. Notably, single-dose anti-CCR7 treatment seemed to delay disease onset to week 8 (as determined by the first appearance of bioluminescent signals) and also tended to modestly reduce tumor burden, although no statistical differences were found between groups (Fig. 5 b,c). Similarly, post-mortem analysis demonstrated that this ‘preventive’ treatment had a mild though non-significant impact on the cell viability (specifically in LN) and proliferation (in LN and other tumor niches like spleen or brain) of SUP-T11-luc^+^ cells (Fig. 5 d). Together, these results suggest that CCR7 might act as a key player in niche colonization and formation by SUP-T11 cells and advance a potential role of anti-CCR7 therapy in impairing the formation of pro-tumorous sanctuaries in T-PLL, such as LN or brain, by reducing migration to tumor niches, and, in addition, by affecting survival and proliferation of tumor cells within these locations.

## Discussion

T-PLL is an aggressive disease with a poor prognosis. Despite allo-HSCT is given under curative intent, disease control for more than 5 years is documented in a minor fraction of patients [1, 17]. Therefore, curative therapies are not available for the majority of cases. In this work, we show that CCR7 is highly expressed on T-PLL cells and that this expression has a negative impact on patient survival. Indeed, in our cohort, CCR7 expression was found in 83 out of 96 cases (~ 86.5%) and patients lacking CCR7 (13.5%) tended to have a better overall prognosis. Nonetheless, our results on CCR7 expression also confirm that T-PLL is a very complex and heterogeneous disease, therefore, further confirmation of the impact of CCR7 in short T-PLL patient survival is needed with standardized flow cytometry protocols in larger cohorts. Even though, this is the first time that such a correlation is presented in blood cancers that makes CCR7 a reasonable therapeutic target in T-PLL.

Our results also suggest that CCR7 is overexpressed in T-PLL when compared to pan-T cells obtained from HD and that expression in T-PLL certainly resembles a transition in the differentiation stages of T-cells, with a vast majority of patients showing CCR7 expression similar to that found in T_N_ and T_CM_ phenotypes. When analyzing these data in the context of an anti-CCR7 therapeutic approach, we could show that (i) a relevant proportion of T-PLL patients would potentially be amenable to this treatment as they strongly express CCR7 and (ii) that one could potentially expect a limited hematologic toxicity since not all normal T-cell subsets of the patient would be targeted (e.g. pathogen-specific CCR7^−^ effector cells).

In addition, we demonstrate CCR7 functionality in the spread of T-PLL. CCR7 is shown to trigger migration and invasion of T-PLL cells and to facilitate an invasive phenotype by promoting the secretion of MMP-9. Our data also suggest that CCR7 enables access of T-PLL T-cells to lymphoid and non-lymphoid organs thus confirming the role of CCR7 guiding hematogenous and lymphatic spread of malignant cells [19, 21, 29, 44–46]. In these localizations, and more specifically in the LN, CCR7 may mediate survival of T-PLL cells upon CCR7 ligand engagement, as suggested by our in vitro and in vivo studies. Accordingly, stromal cells in the LN may confer resistance to spontaneous apoptosis by secreting CCL19 and IL-7 [47]. In addition, CCR7 may mediate proliferative cues on T-PLL cells. Although we do not show a direct impact of CCR7 blockade on in vitro proliferation, we cannot exclude that leukemic cells guided via CCR7-ligand based chemotaxis to protective niches (e.g. brain, spleen and LN) could find other proliferative stimuli such as soluble factors or other cell types, including dendritic cells or stromal cells [21, 22, 43, 48]. Indeed, we show that targeting CCR7 has an impact on proliferating Ki67^+^ tumor cells in vivo.

In T-PLL, alemtuzumab responses are transient and disease progression and acquisition of resistance are inevitable [1]. Therefore, alternative treatment strategies are urgently needed. In this work we also evaluated mAb-based anti-CCR7 therapy as a novel tool of treatment in this disease. Our findings support a double mechanism of action of this type of antibodies consisting in the combination of cell killing and blocking of cell spread and niche homing (Supplementary Figure 7). Anti-CCR7 mAbs killed leukemic cells derived from both untreated and alemtuzumab-refractory patients through host effector mechanisms, specifically CDC. Also, the anti-CCR7 mAb used in this study has an additional mechanism to induce T-PLL cell death, namely by blocking the interaction of CCR7 with its ligands and thus disrupting pro-survival pathways. We did not specifically study which signaling molecules are involved in this process, however, blocking CCR7 abrogated PI3K and ERK activation, two important components of CCR7-mediated pro-survival pathways in hematological malignancies [23, 35] that have shown to be relevant in T-PLL pathogenesis [49, 50]. In T-PLL, disease distribution is an important factor determining outcome. For example, the proportion of cases that respond to alemtuzumab is low in patients with CNS disease [1] and/or bulky LN masses [8, 51]. In this regard, anti-CCR7 showed here to be effective in eradicating tumor cells from several localizations, including LN and CNS. Anti-CCR7 therapy also inhibited secretion of MMP-9 and blocked T-PLL cell invasion in matrices and through endothelia. Targeting these processes is important to block lymphatic and distant dissemination of tumor cells as inferred from several clinical studies on T-cell lymphomas, chronic lymphocytic leukemia, or solid tumors [19, 24, 29, 46, 52]. Indeed, it is worth highlighting that multiple prominent high endothelial veins (HEV) are often infiltrated by neoplastic cells in T-PLL [53], which suggests CCL21 as a major route for homing into lymphoid tissues and mediating the dissemination of T-PLL cells to different organs. Accordingly, results obtained from our novel in vivo models of T-PLL confirm that blocking CCR7 might be a relevant way to impair homing of leukemic T-cells to protective niches. Consistent with this action, anti-CCR7 mAbs tended to delay disease onset, most likely by the inhibition of CCR7-guided niche occupation. Similarly, Rhem et al demonstrated that engraftment of CCR7-deficient lymphoma B-cells was associated with a significant delay in the appearance of disease [43].

Overall, our results envision anti-CCR7 mAbs as a promising therapeutic application for T-PLL, a disease where antibodies were not included in recent searches for novel promising drugs. Particularly, anti-CCR7 mAbs could be beneficial in the context of alemtuzumab-relapsed/refractory disease or in patients with low-level CD52 expression [8, 36] or lack of CD52 [54, 55]. According to our data, anti-CCR7 therapy could also target or avoid CNS infiltration, tackle bulky lymphadenopathies, or prevent T-PLL relapse in settings of minimal residual disease in lymphoid organs or other sites where alemtuzumab is ineffective in tumor cell clearance. To this end, anti-CCR7 mAbs could be used in monotherapy or combined with alemtuzumab, with purine analogs, or with novel small-molecule inhibitors that are under development and that target other, potentially synergistically acting pathways [56–59]. Finally, targeting CCR7 with mAbs may fulfill not only the urgent need for more rationally based therapies in T-PLL, but also in many other CCR7-expressing T-cell malignancies such as SS/MF [20, 60], T-ALL [21, 22], and ATLL [19, 61], in which particularly aggressive diseases characteristically express high levels of CCR7 [19, 61] and where few standard mAb-based treatment strategies have been developed so far.

## Conclusions

In this report, we have described for the first time the role of CCR7 in T-PLL. The activity of this receptor associates to clinical outcome and contributes to a widespread dissemination of the disease to specific niches where leukemic cells find proliferative and survival cues. Moreover, our results on the activity of an anti-CCR7 mAb, both in vitro and in vivo confirm CCR7 as an attractive molecule for novel mAb-based therapeutic applications in T-PLL, a disease where recent drug screen efforts and studies addressing new compounds have focused on chemotherapy or small molecules.

## Supplementary information

**Additional file 1.** this file provides additional information on the methods and supplementary figures.

## Data Availability

The datasets used and/or analyzed during the current study are available from the corresponding author on reasonable request.

## Abbreviations

ADCC: Antibody-dependent cell-mediated cytotoxicity
Allo-HSCT: Allogeneic hematopoietic stem cell transplantation
ANOVA: Analysis of variance
ATLL: Adult T-cell leukemia/lymphoma
BM: Bone marrow
CCR7: C-C chemokine receptor 7
CCL19/21: C-C-chemokine ligand 19/21
CD: Cluster of differentiation
CDC: Complement-dependent cytotoxicity
CNS: Central nervous system
Fab: Fragment antigen-binding
Fc: Fragment crystallizable
FcγR: Fc gamma receptor
HD: Healthy donor
HEV: High endothelial veins
HR: Hazard ratio
HUVEC: Human umbilical vein endothelial cells
IC: Isotype control
IL-7: Interleukin 7
iv: Intravenously
LN: Lymph node
mAb: Monoclonal antibody
MEK/ERK1/2: Mitogen-activated protein kinase kinase / extracellular signal-regulated kinase
MOA: Mechanism of action
MMP: Matrix metalloproteinase
MTCP1: Mature T Cell Proliferation 1
NK: Natural killer
NSG: NOD.Cγ-Prkdcscid-IL2rgtm1Wjl/SzJ mice
OS: Overall survival
PB: Peripheral blood
PBMCs: Peripheral blood mononuclear cells
PI3K: Phosphatidylinositol 3-kinase
RMFI: Relative median of fluorescence intensity
ROCK/MLC: Rho-associated protein kinase / myosin light chain
R/R: Relapsed/refractory
SD: Standard deviation
SEM: Standard error of the mean
SLO: Secondary lymphoid organs
SS/MF: Sézary syndrome / Mycosis Fungoides
T-ALL: T-cell acute lymphoblastic leukemia
TCL1: T-cell leukemia/lymphoma 1
T_CM_: Central memory T-cells
T_N_: Naïve T-cells
TEM: Trans-endothelial migration
TME: Tumor microenvironment
T-PLL: T-cell prolymphocytic leukemia
