# Supplementary material for: CCR7 as a novel therapeutic target in t-cell PROLYMPHOCYTIC leukemia
Source: Biomark Res. 2020 Oct 24;8:54. doi: 10.1186/s40364-020-00234-z (PMC7585232; doi:10.1186/s40364-020-00234-z)
Supplement: Supplementary file 1 — Additional file 1. this file provides additional information on the methods and supplementary figures. [file 40364_2020_234_MOESM1_ESM.docx]

**SUPPLEMENTARY MATERIAL AND METHODS**

**Actin polymerization**

T-PLL cells (5 x 10^5^) were suspended in 100 µl RPMI-1640 + 0.1% BSA and incubated at 37°C with 1 µg/mL recombinant CCL19 or CCL21 for different times. Cells were then fixed-permeated using the lysing solution (BD Biosciences) according to the recommendations of the manufacturer. For staining actin filaments, cells were incubated with 5 µg/mL Alexa-647-phalloidin (Molecular Probes, Eugene, OR) and analyzed by flow cytometry. Results are expressed as the percentage of intracellular F-actin relative to the value found in untreated cells (t=0).

**Immunofluorescence microscopy**

#### To determine cell shape rearrangement involved in migration, ICAM-3 polarization assays were performed by microscopy. Cells were plated on glass coverslips previously coated with fibronectin. Then, cells were starved for 1 h and stimulated with CCL19 or CCL21 (1 µg/mL) for different times. Anti-CCR7 mAb was used to block CCR7-ligands interaction. Then cells were fixed in ice-cold paraformaldehyde and permeated using a 0.2% Triton-X-100 solution. After washing and blocking, cells were sequentially labeled with primary antibodies against ICAM-3 (clone TP1/25, provided by Dr. Sánchez-Madrid) and with fluorescent-tagged species-specific secondary antibodies. Images were acquired on a Leica-DMR fluorescent microscope (Leica, Wetzlar, Germany) equipped with a Leica-DFC360FX-camera using Leica Application Suite V.3 and processed with the ImageJ (NIH) software.

**β-arrestin recruitment assay**

#### Antagonism was evaluated in established PathHunterTM β-arrestin recruitment assays, (DiscoverX, Fremont, CA). Tests were conducted following manufacturer’s protocols with hCCR7+ CHO-K1 cells exposure to antagonists before 80% effective concentration (EC80) of agonist was added. Antagonism was calculated with: Inhibition (%) =100 x [1 - (signal test - signal vehicle) / (signal ligand - signal vehicle)].

#### Internalization assay

#### Internalization of membrane-expressed CCR7 was assayed by flow cytometry in CLL cells as described by Alfonso-Pérez et al ([1](#_ENREF_1)).

**SUPPLEMENTARY FIGURES**

**SUPPLEMENTARY FIGURE 1**

**
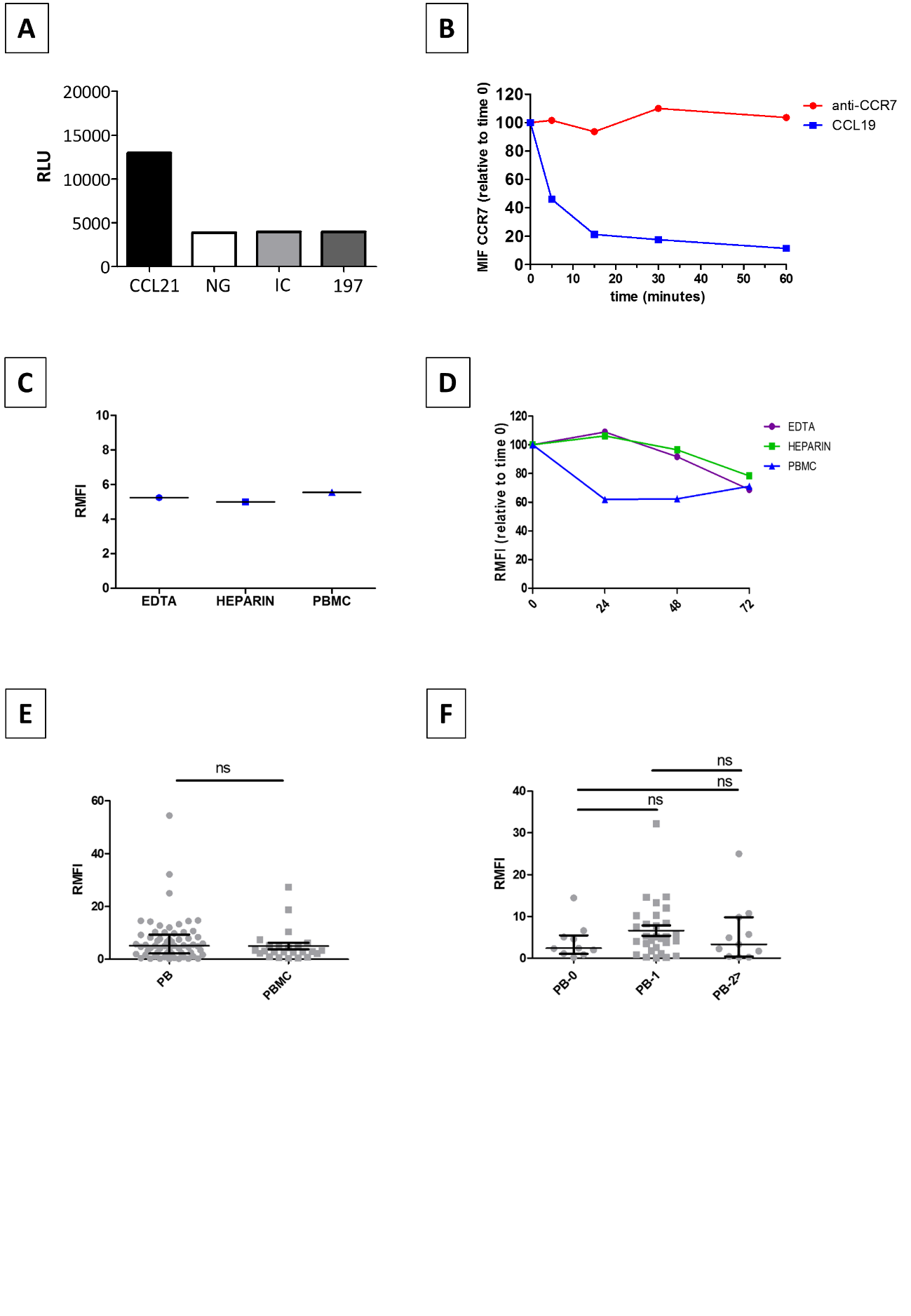
**

**Supplementary Figure 1. A-B) Anti-CCR7 (197; clone 150503) has no agonistic effects and does not induce target internalization. A)** At high, saturating concentrations (40 µg/ml), the anti-CCR7 antibody did not induce detectable intracellular agonistic effects in one β-arrestin recruitment assay. Stably transfected hCCR7^+^ CHO cells were used as target cells in one single experiment. The graph shows the relative luminescence units (RLU) for each condition. Matched isotype controls IgG2a (IC), and medium alone (NG) were used as negative controls, and CCL21 (50 nM) as a positive control. **B)** The clone 150503 does not induce CCR7 internalization in T-PLL cells (n=1). Flow cytometry analysis in primary T-PLL cells (n=1) incubated with a 10 μg/ml of antibody or 1 μg/ml of CCL19 for the indicated times. The CCR7 surface expression, measured as the median fluorescence intensity (MFI), is normalized to time 0. The ligand CCL19 was used as a positive control as it is largely known to induce a strong CCR7 internalization ([2](#_ENREF_2)). As seen in the figure, the anti-CCR7 antibody used did not affect the expression of CCR7 during the expected time of sample incubation**.**

**SUPPLEMENTARY FIGURE 2**

**
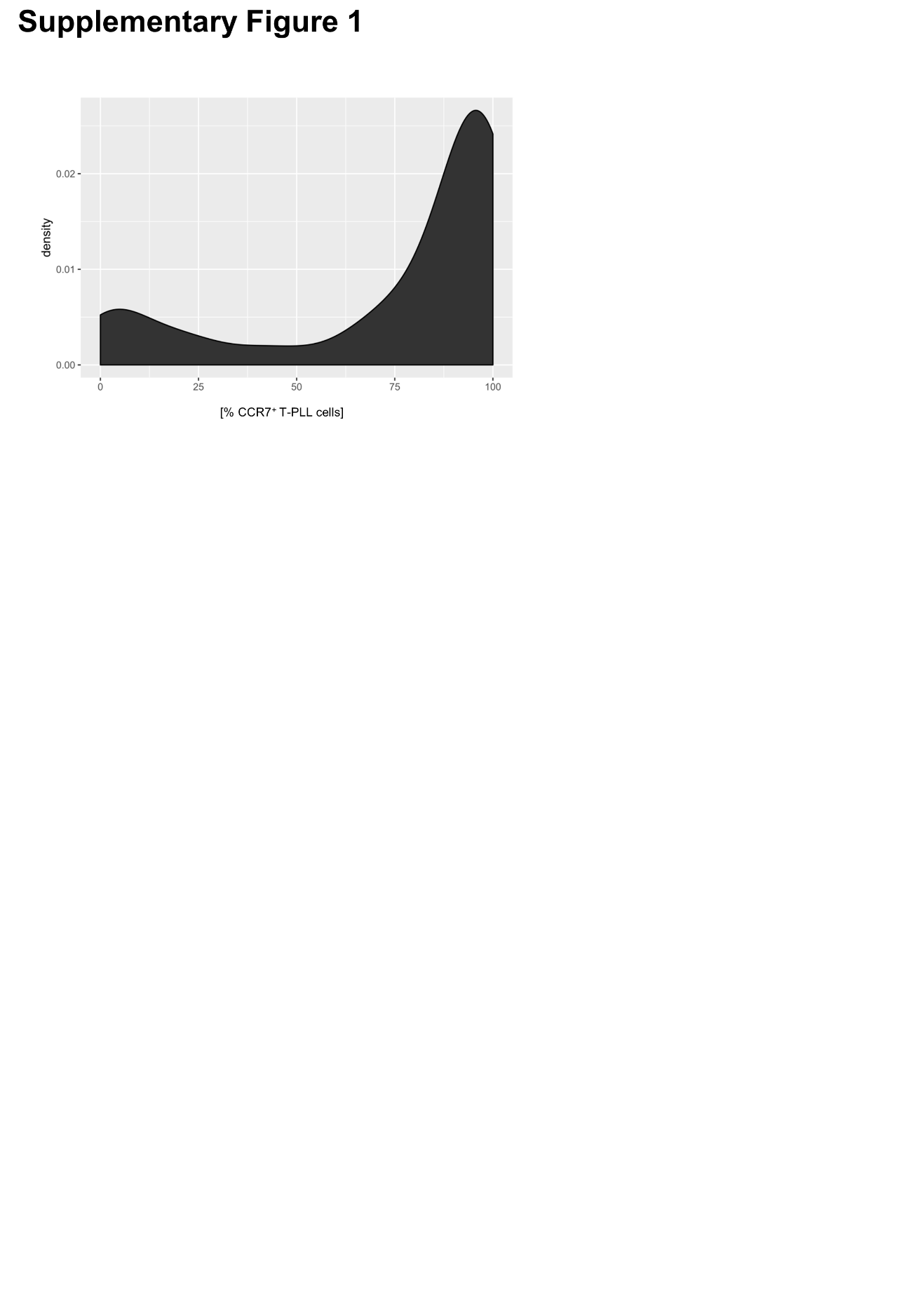
**

**Supplementary Figure 2. Density distribution of CCR7 positivity in T-PLL patients.** The graph shows the distribution of patients (n=97 T-PLL cases) according to the percentage of CCR7-positive T-PLL cells detected via flow cytometry in peripheral blood samples.

**SUPPLEMENTARY FIGURE 3**


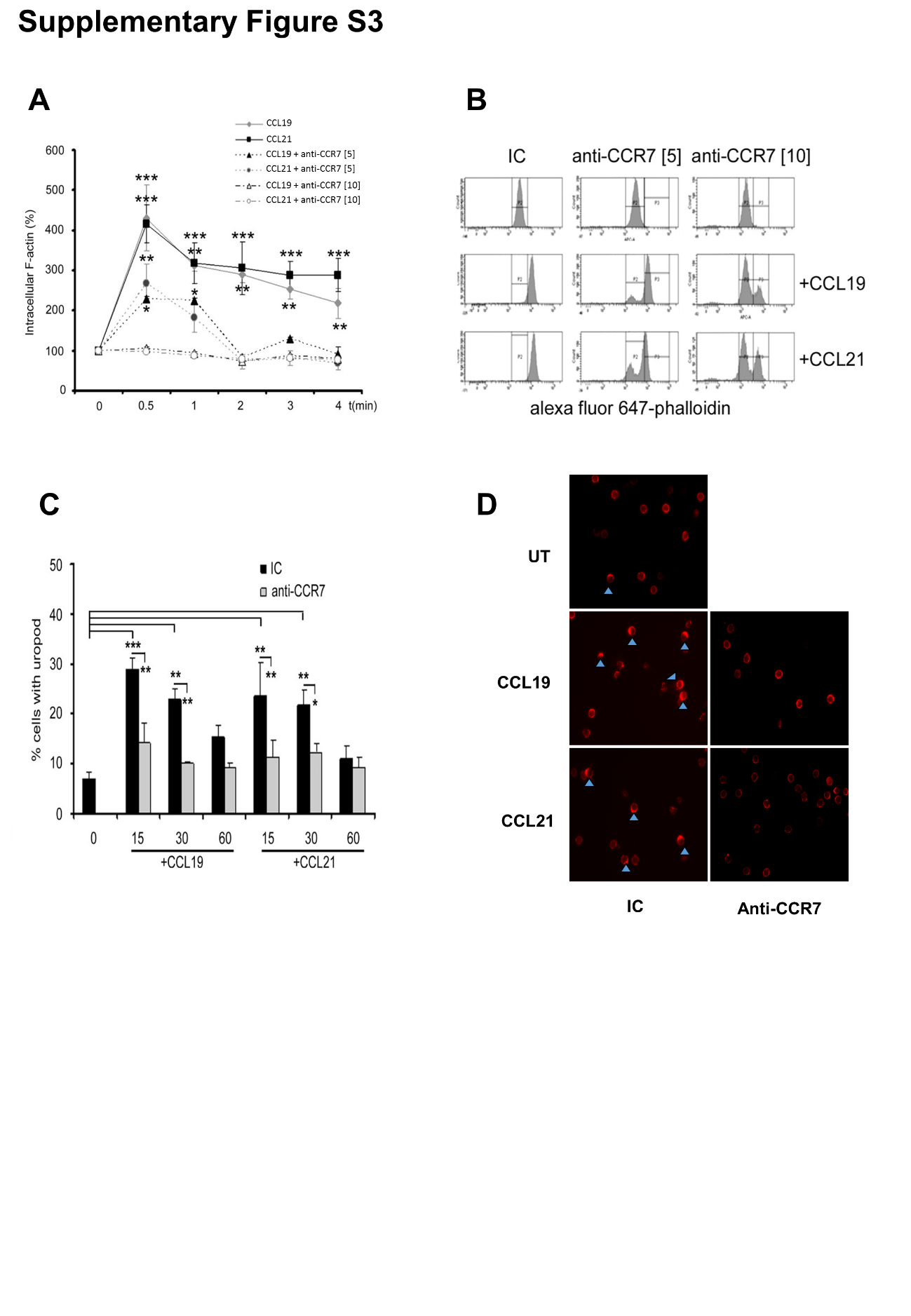


**Supplementary Figure 3**. **CCR7 is functional in T-PLL cells. A-B) CCR7 ligands induce actin polymerization, a process inhibited by anti-CCR7 mAb. A)** After the binding of ligands, a significant increase of intracellular F-actin was observed, peaking at 0.5 min with both chemokines and recovering basal levels along the time. In addition, we observed that actin polymerization was inhibited in a dose dependent manner when cells were incubated with anti-CCR7 mAb prior to adding the chemokines. Actin polymerization was determined by flow cytometry by means the staining of F-actin with Alexa Fluor 647-phalloidin. To this end, T-PLL cells were incubated with chemokines (1 µg/mL) at 37°C for different periods of time. Reorganization of the actin cytoskeleton in response to CCR7 ligands was quantified measuring the median fluorescence intensity relative to t=0 (non-stimulated cells) as described in Materials and Methods. An increase of alexa-fluor-647 phalloidin was identified as actin polymerization. An early initial response was observed with both chemokines which was partially impaired with anti-CCR7 mAb at 5 µg/mL or completely abrogated at 10 µg/mL. Data represent the means derived from five separate experiments ± SEM. **B)** A representative case of experiments described in **A** showing actin polymerization in T-PLL cells. Cells were incubated for 0.5 min with CCL19 or CCL21. The displacement to the right in chemokine-stimulated cells indicates actin polymerization. In cells pre-incubated with anti-CCR7 mAb at 5 or 10 µg/mL the number of events showing actin polymerization decreased proportionally to the anti-CCR7 mAb concentration. **C-D) CCR7 ligands induce ICAM-3 clustering in cellular uropods of T-PLL cells.** Another process that takes place during chemokine-dependent lymphocyte activation and migration is the development of a well-defined cytoplasmic projection designated as cellular uropod. This projection is a structure necessary for an efficient chemotactic migratory response where the ICAM-3 adhesion molecule (a ligand for LFA-1) is concentrated. When spherical-shaped resting T-PLL cells were stimulated with CCL19 and CCL21 they become polarized and ICAM-3 clustered in uropods. Approximately one third of T-PLL cells displayed such a migratory phenotype fifteen min after the addition of the chemokines which was abrogated with anti-CCR7 mAb. **C)** Quantification of cells showing ICAM-3 at uropods after CCL19/CCL21 treatment**.** One hundred cells of each condition where scored and analyzed. Treatment with chemokines increased the number of cells presenting cellular uropods compared to non-stimulated cells (black bars). This effect was highly reduced when CCR7 was previously blocked with an anti-CCR7 mAb (10 µg/mL; grey bars). Data represent the means derived from five separate experiments. Error bars represent SEM. **D)** Leukemic cells were left untreated (UT) or were stimulated with 1 µg/mL of chemokines (only t= 15 min is shown). Then, cells were fixed and stained for ICAM-3 (clone TP1/25), red fluorescence. Arrows denote immunofluorescence localization of ICAM-3 positive uropods. When cells were treated with a mAb against CCR7 prior to ligand addition, the effect mediated by CCR7 was strongly impaired (right column). Images shown are representative images from five independent experiments. IC, isotype control. *, p <0.05; **, p <0.01; ***, p <0.001; ns, not significant.

**SUPPLEMENTARY FIGURE 4**

**
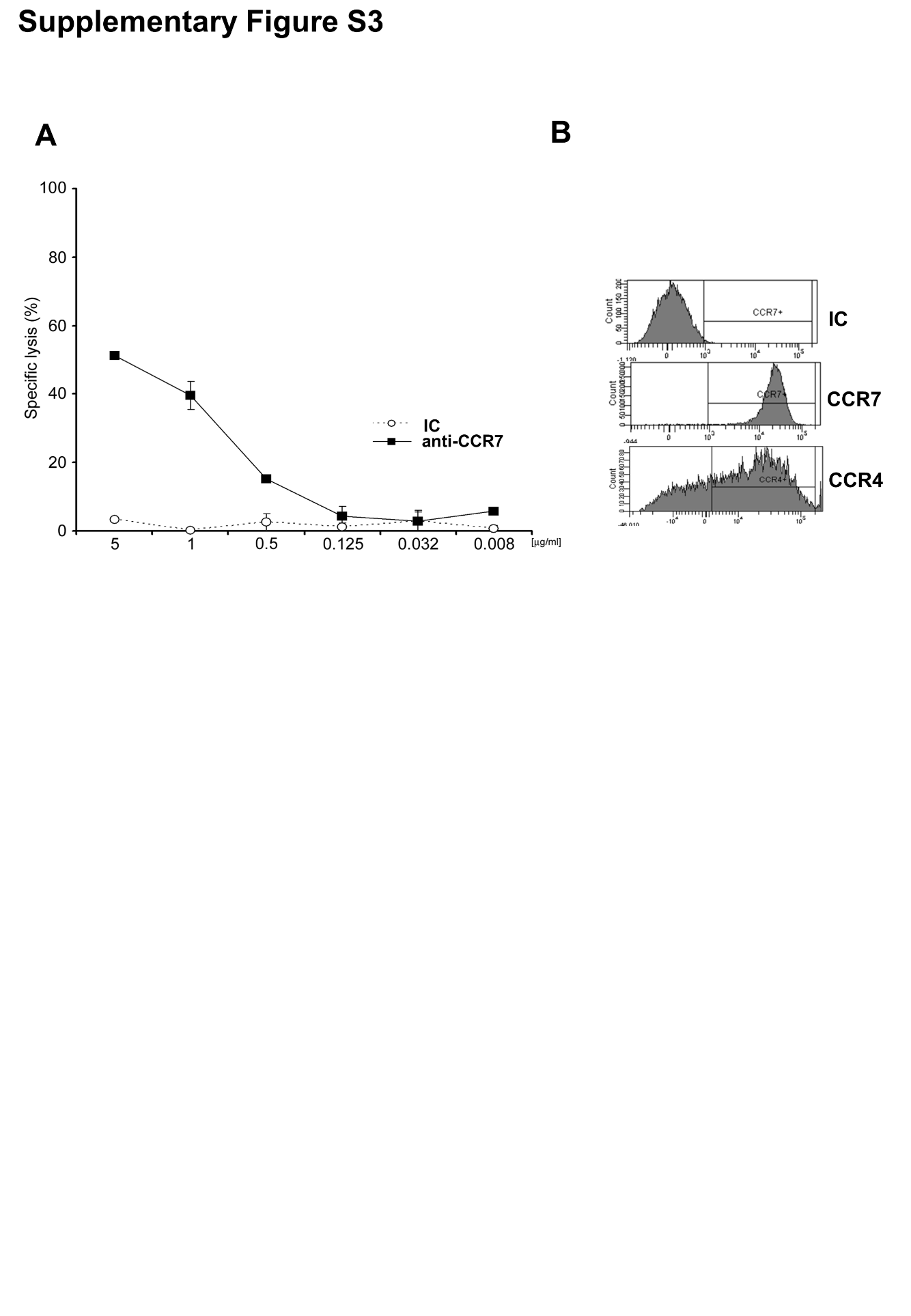
**

**Supplementary Figure 4**. **A) Anti-CCR7 mediates CDC in Sézary Syndrome (SS) cells. A)** The percentage of specific lysis in CD4^+^CD7^-^ SS cells is shown in this figure. PBMC from SS patients (n=2) were incubated with different concentrations of anti-CCR7 or the respective IC in the presence of 25% of either active or heat-inactivated rabbit complement. Percentage of cell lysis as a result of CDC was determined according to the formula stated in Material and Methods by 7-AAD incorporation and flow cytometry analysis in gated SS cells. Each square or triangle represents mean ± SEM. **B) Comparative analysis on the expression of CCR4, the target of mogamulizumab, and CCR7 in SS cells.** Frequency histograms showing the pattern and intensity of CCR7, CCR4 and the appropriate isotype control (IC) in a representative SS patient are shown.

**SUPPLEMENTARY FIGURE 5**


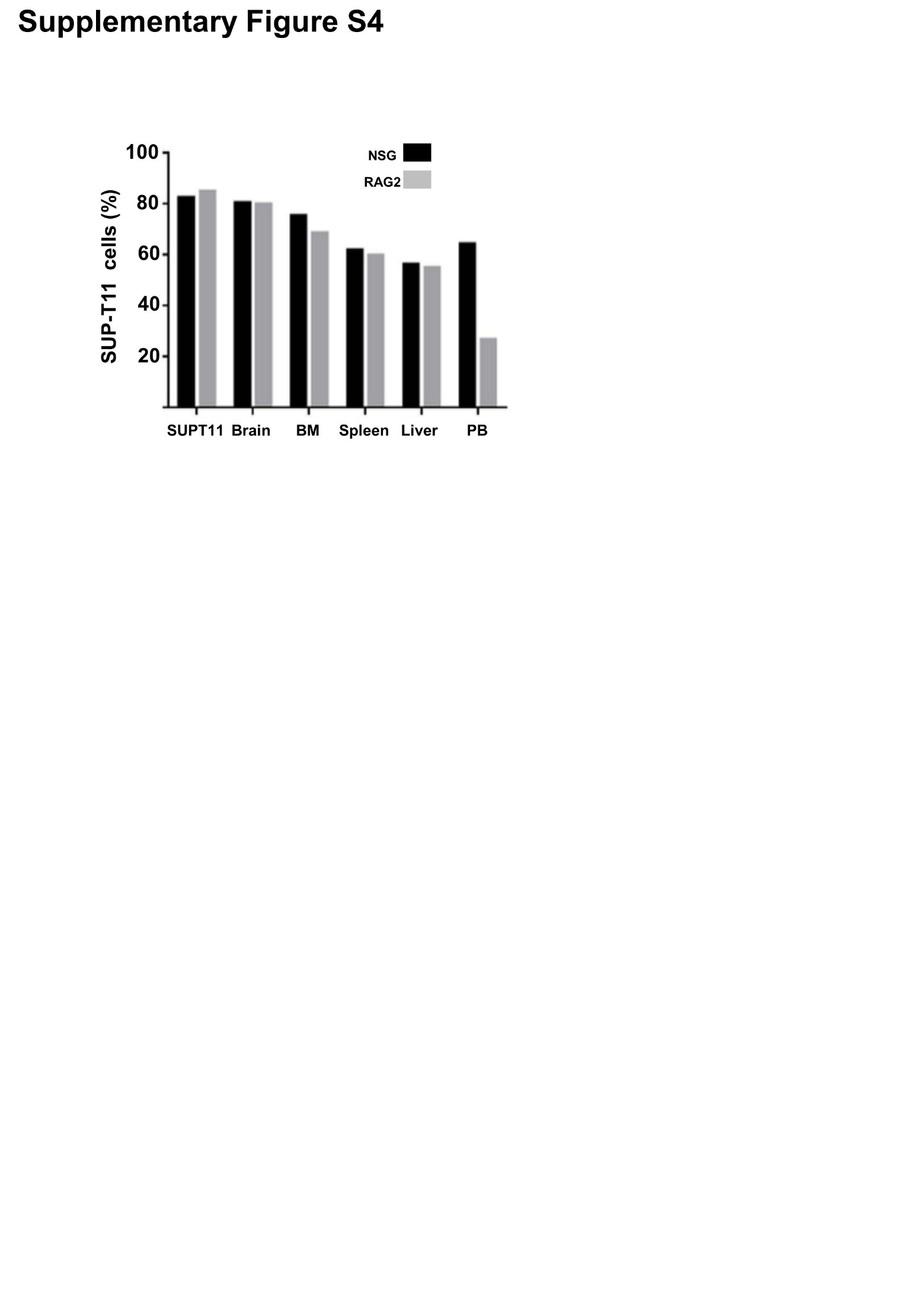


**Supplementary Figure 5**. **SUP-T11 cells engraft in immunodeficient mice.** Tumor cells were injected in the vein of NSG (black columns) or RAG2^-/-^γc^-/-^ (grey columns) mice. After the sacrifice, different organs were disaggregated and cell suspensions were stained with anti-hCD45 and anti-hCD5. The percentage of SUP-T11-CCR7^+^ cells in each organ is shown.

**SUPPLEMENTARY FIGURE 6**


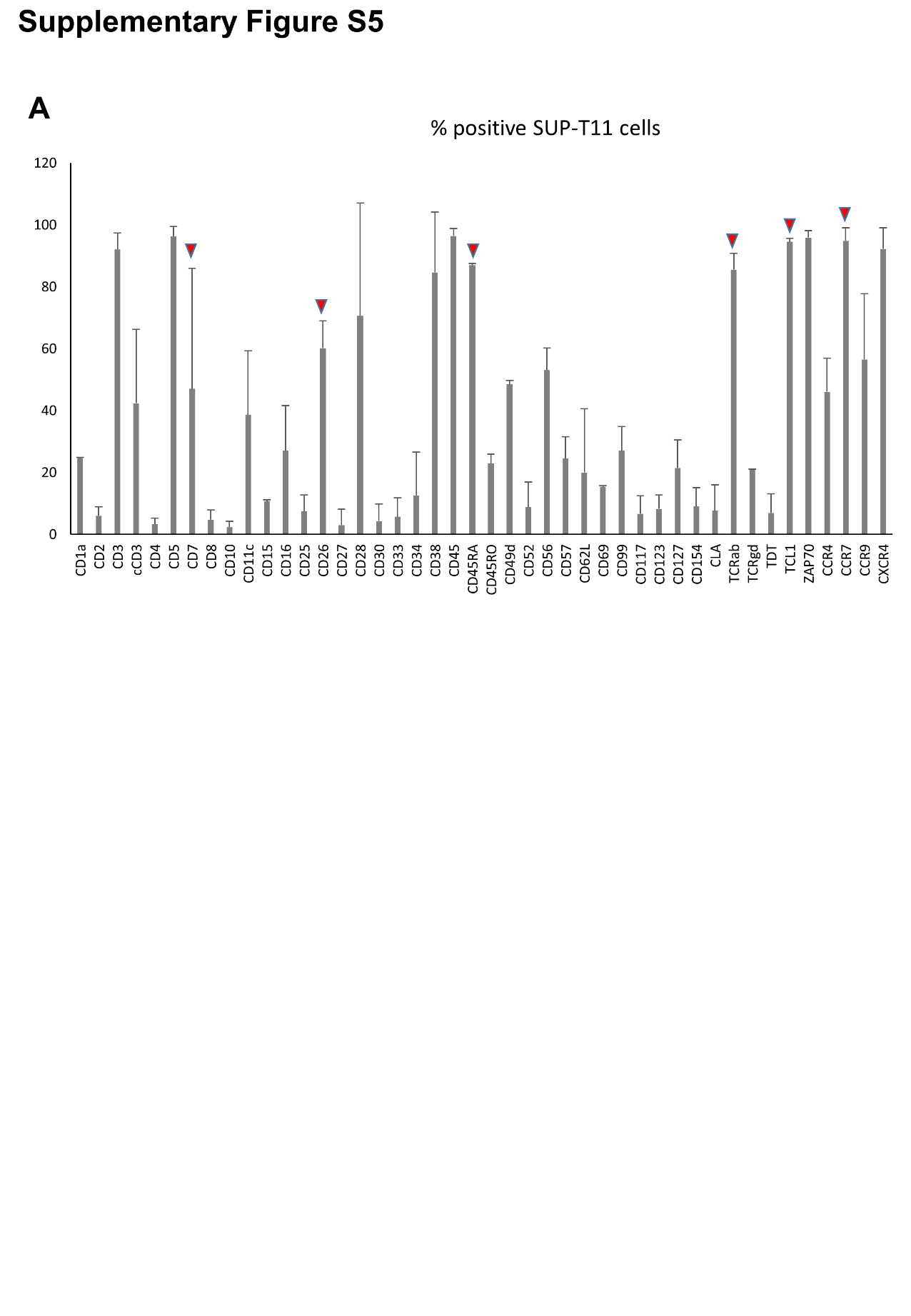


**Supplementary Figure 6**. **Phenotype of SUP-T11 cells.** Tumor cells were stained with commercial antibodies targeting several surface and intracellular markers including lineage antigens, markers related to T-cell activation and chemokine receptors. **A)** The percentage of positive SUP-T11 cells is shown for each marker. A single marker was considered positive when the proportion of positive cells was higher than 20% as described by Bain *et al.*([3](#_ENREF_3)).

**SUPPLEMENTARY FIGURE 7**


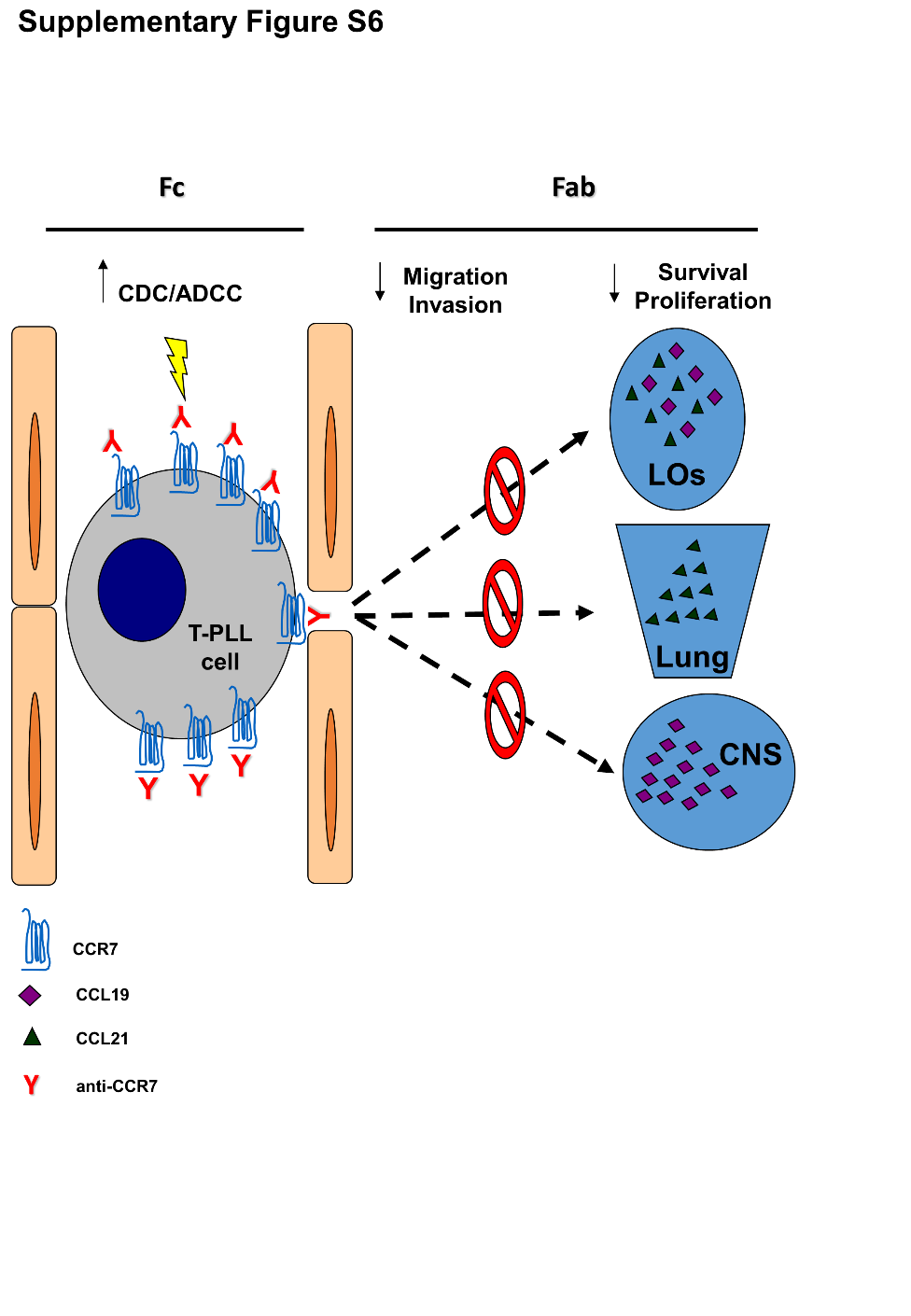


**Supplementary Figure 7**. **Anti-CCR7 therapy is effective in T-PLL through a double mechanism of action (MOA).** In T-PLL, CCR7 mediates migration and invasion to lymphoid and non-lymphoid organs where CCR7 directly mediates pro-survival effects. Indirectly, CCR7 may participate in guiding leukemic cells to these sanctuaries where cells might find several proliferative stimulus. Anti-CCR7 mAb therapy is presented as a novel and potential tool in the treatment of T-PLL owed to its double and effective MOA: it kills leukemic cells through effector mechanisms and indirectly by blocking pro-survival signaling pathways. Furthermore, it blocks leukemic cells migration and invasion which can be relevant at avoiding homing of leukemic cells to TME of lymphoid and non-lymphoid organs where these cells can proliferate or escape from other therapeutic agents. Additionally, anti-CCR7 antibodies may increase the number of cells in PB which can be killed by immune effector mechanisms or other drugs present in the PB.

**SUPPLEMENTARY REFERENCES**

1. Alfonso-Perez M, Lopez-Giral S, Quintana NE, Loscertales J, Martin-Jimenez P, Munoz C. Anti-CCR7 monoclonal antibodies as a novel tool for the treatment of chronic lymphocyte leukemia. J Leukoc Biol. 2006;79(6):1157-65.

2. Hauser MA, Legler DF. Common and biased signaling pathways of the chemokine receptor CCR7 elicited by its ligands CCL19 and CCL21 in leukocytes. J Leukoc Biol. 2016;99(6):869-82.

3. Bain BJ, Barnett D, Linch D, Matutes E, Reilly JT. Revised guideline on immunophenotyping in acute leukaemias and chronic lymphoproliferative disorders. Clin Lab Haematol. 2002;24(1):1-13.
